# High‐permeability cellulose nanocrystals mediate systemic zinc redistribution through nsLTP2‐dependent immune potentiation in plants

**DOI:** 10.1111/pbi.70230

**Published:** 2025-06-26

**Authors:** Jing Wang, Shunyu Xiang, Xiaoyan Wang, Yang Shen, Changyun Liu, Xin Zhu, Weina Liu, Shanzhi Wang, Xiaozhou Ma, Jin Huang, Xianchao Sun

**Affiliations:** ^1^ College of Plant Protection Southwest University Chongqing China; ^2^ Chongqing Key Laboratory of Soft‐Matter Material Chemistry and Function Manufacturing Southwest University Chongqing China

**Keywords:** zinc, cellulose nanocrystal, antivirus, plant immunity, TMV

## Abstract

Zinc (Zn^2+^) is an essential micronutrient that regulates plant growth, immunity and antiviral defence mechanisms. However, its limited bioavailability often necessitates excessive application, resulting in inefficiencies in production and environmental stress. In response, we propose an environmentally friendly and sustainable approach to enhance the utilization of Zn^2+^. We developed CNC@PDA@Zn^2+^ by embedding Zn^2+^ into the polydopamine (PDA) coating of cellulose nanocrystals (CNCs). Leveraging the high cell permeability of CNCs, this material increased the transport capacity of Zn^2+^ in plants and demonstrated the ability to inactivate viral particles *in vitro*. Moreover, CNC@PDA@Zn^2+^ showed a superior induction of resistance while reducing Zn^2+^ content, specifically by reprogramming the expression and localization of the resistance‐related non‐specific lipid transfer protein 2 (nsLTP2), which enhanced the salicylic acid (SA) signalling pathway in plants. Furthermore, the high conservation of nsLTP2 in flowering plants increases the potential application range of CNC@PDA@Zn^2+^. Importantly, CNC@PDA@Zn^2+^ represents the most effective Zn^2+^‐based antiviral nanomaterial to date, achieving its effects at the lowest reported Zn^2+^ concentration. Overall, our results highlight that CNC@PDA@Zn^2+^ can more effectively upregulate the conserved nsLTP2, thereby inducing viral defence responses via the SA pathway. This strategy not only improves the operation and utilization rate of Zn^2+^ but also reduces its environmental residues, laying a theoretical foundation for the development of antivirus defence.

## Introduction

Plant viruses pose a significant threat to agriculture, causing substantial reductions in crop quality and yield upon infection (Jones and Naidu, 2019). The tobacco mosaic virus (TMV), recognized as one of the most detrimental plant viruses, has been identified to infect over 350 host plant species, including economically important crops such as tobacco, tomatoes and peppers, leading to substantial economic losses (Scholthof *et al*., 2011). Upon infection, TMV disrupts chloroplasts within plant cells, impedes photosynthesis and results in mottled and discoloured leaves, while also affecting cell division and causing leaf deformities (Guo *et al*., 2015). Currently, there is no agent capable of fully eradicating TMV (Jiang *et al*., 2024). The prevention and management of viral diseases associated with TMV largely rely on the use of viral inhibitors, which face challenges such as limited storage stability, environmental pollution, excessive pesticide residues and the development of pathogen resistance (An *et al*., 2022b). With advances in understanding plant immune mechanisms, there has been a shift towards utilizing plant immune inducers for disease prevention and control. These inducers trigger immune responses, including reactive oxygen species (ROS) accumulation, calcium (Ca^2+^) signalling, callose deposition, salicylic acid (SA) production and the expression of resistance‐related genes (Lee *et al*., 2020; Xiang *et al*., 2023b; Yang *et al*., 2022b; Zeng *et al*., 2024), ultimately leading to systemic acquired resistance (SAR). During SAR, SA levels increase, and resistance genes such as pathogen‐related protein (PR) genes are upregulated (Jones and Dangl, 2006; Kachroo and Kachroo, 2007; Yang *et al*., 2022a; Zhou and Zhang, 2020).

The utilization of zinc (Zn^2+^) for disease control dates back to as early as 1938 and potentially even earlier (Callbeck, 1954; Millikan, 1938). Zn^2+^, an essential micronutrient for plant growth, is integral to various biological processes, including metabolic pathways, enzymatic reactions, hormone metabolism, disease resistance and stress tolerance. More recently, it has been recognized for its role in the regulation of nitrogen fixation efficiency within root nodule symbiosis (Auld, 2001). As previously highlighted, plant immunity against pathogens is associated with SA accumulation in non‐infected tissues, ROS burst and the initiation of SA‐dependent SAR. In this context, C9 dicarboxylic acids and azelaic acid (AzA) are known to participate in the long‐distance signalling of SA (Breitenbach *et al*., 2014). It is significant that under Zn^2+^‐rich and AzA conditions, the expression of *PR1* is more readily upregulated compared with Zn^2+^‐deficient conditions, indicating that adequate Zn^2+^ levels can enhance the production of resistance‐related metabolites, thereby influencing plant resistance levels. Prior research has shown that the lipid transfer protein‐like protein encoded by *AtAZI1* (Azelaic acid induced 1) is involved in transmitting SAR signals in response to AzA, thereby activating local or systemic non‐specific resistance to pathogens (Jung *et al*., 2009), and this function of AtAZI1 is Zn^2+^‐dependent (Nadia *et al*., 2018), establishing a link between plant nutritional immunity and micronutrient utilization. Moreover, the application of Zn^2+^ fertilizers under drought conditions has been shown to increase antioxidant content (e.g. ascorbic acid, reduced glutathione, total flavonoids, total phenols) in wheat leaves, and foliar Zn^2+^ spraying can enhance photosynthetic pigments, reduce lipid peroxidation of cell membranes, maintain cellular homeostasis and mitigate drought‐induced oxidative damage (Hamzah Saleem *et al*., 2022). Thus, Zn^2+^ emerges as a potent plant immune inducer and growth regulator (Cakmak *et al*., 2010; Ma *et al*., 2017).

Despite the numerous studies highlighting Zn^2+^'s role in pathogen control, the application of Zn^2+^ fertilizers still tends to be at relatively high concentrations (Awan *et al*., 2019). Due to environmental complexities, the loss of active ingredients during the application of trace elements can reach 70%–90% (Deng *et al*., 2016), which compromises the maximum absorption of Zn^2+^ by plants (Zhang *et al*., 2020). Repeated applications not only increase economic costs but also impose environmental stress, leading to plant stress (Broadley *et al*., 2007; Kheyri and Taheri, 2021). In our previous study, we determined that 44 μg/mL of (CH_3_COO)_2_Zn (Zn^2+^content is 237.3 μM) exhibited antiviral properties while still allowing normal plant growth (Wang *et al*., 2022a). Furthermore, Zn^2+^ toxicity symptoms have been reported to manifest at concentrations of 300 mg/kg leaf dry weight, although some crops have lower thresholds (Broadley *et al*., 2007). These findings underscore the high loss rates of Zn^2+^ in practical applications and the potential environmental risks associated with its use. To address these challenges, we implemented various delivery strategies to mitigate Zn^2+^ loss (Cao *et al*., 2023; Zhu *et al*., 2024a). In addition, other research groups have sought to enhance Zn^2+^ utilization, primarily through the development of soil or foliar fertilizers based on nanostructured materials. For instance, foliar application of 20 mg/L ZnO nanoparticles (ZnO‐NPs) significantly promoted the growth of mung bean (*Vigna radiata* (L.) R. Wilczek) and chickpea (*Cicer arietinum* L.) compared with conventional fertilizers (Mahajan *et al*., 2011). Moreover, ZnO‐NPs resulted in greater Zn^2+^ accumulation in the roots of *Schoenoplectus tabernaemontani* than Zn^2+^ solutions (Zhang *et al*., 2015). However, more optimized delivery methods are still required to minimize Zn^2+^ residues in the environment and enhance the efficiency of Zn^2+^ application (Zhao *et al*., 2022).

Due to the size effect and high permeability of nanomaterials, they can effectively enhance the stability of pharmaceutical ingredients, facilitate drug deposition on the application interfaces and improve drug utilization (Zhu *et al*., 2024b). Therefore, they are ideal for drug delivery in the development of plant disease prevention and control agents (An *et al*., 2022a). Rod‐shaped nanomaterials, such as cellulose nanocrystals (CNCs), offer significant advantages as drug carriers for plant disease control due to their high surface area and aspect ratio (Xiang *et al*., 2024; Zhang *et al*., 2011). This feature facilitates cellular binding and uptake while minimizing cytotoxicity, and enhances deposition and retention on the surface of foliage (Park *et al*., 2009; You *et al*., 2022). For instance, surface modification with polydopamine (PDA) enhances CNC's affinity for cells. Additionally, CNCs can adsorb nanoparticles, such as silver, improving their bactericidal effect by increasing particle concentration on bacterial membranes (Drogat *et al*., 2011). Meanwhile, agricultural wastes such as rice husk and straw are rich sources of cellulose nanocrystals, providing a sustainable resource for bio‐composite fabrication (Singh *et al*., 2025). Owing to their plant‐based origin, cellulose nanocrystals are considered highly safe and have found broad applications in both the food and pharmaceutical sectors (Choque‐Quispe *et al*., 2022). Moreover, the properties of CNCs can be enhanced, such as sustaining control, via modification of the CNCs surface (Ning *et al*., 2023; Schiavi *et al*., 2021). For example, acid‐hydrolyzed CNCs demonstrate enhanced thermal and mechanical stability relative to their untreated CNCs (Torlopov *et al*., 2018; Xie *et al*., 2019). Thus, it is reasonable to consider CNCs as pesticide carriers with high deposition efficiency, sustainable pesticide release, and effective antifungal and insecticidal capabilities (Asri *et al*., 2018; Xiang *et al*., 2023a). Based on these analyses, we hypothesize that Zn^2+^ functions as a signalling messenger during plant virus infection. Notably, the Zn^2+^ concentration within plant cells is typically maintained at a steady state of 10–100 nM, while concentrations above 100 μM can induce cellular Zn^2+^ toxicity. Our previous determination of the lowest antiviral concentration of (CH_3_COO)_2_Zn at 44 μg/mL, corresponding to 237.3 μM Zn^2+^, exceeds this threshold. However, increasing the concentration of (CH_3_COO)_2_Zn tenfold demonstrated enhanced resistance without inducing Zn^2+^ toxicity (Wang *et al*., 2022a). This observation challenges the presumed upper limit of the capacity of Zn^2+^ transporters in plants and underscores the substantial loss of (CH_3_COO)_2_Zn during resistance induction. Nonetheless, residual Zn^2+^, if not assimilated by the plant, poses environmental risks. Therefore, it is critical to reduce the applied concentration of Zn^2+^ while enhancing its cellular uptake to optimize its role as a second messenger. Besides, it has been reported that ZnO nanoparticles at concentrations up to 800 mg/kg show no toxicity to cucumber growth which the Zn accumulation in cucumber fruits was 11.1 mg per 100 g of dry cucumber (Zhao *et al*., 2013). By contrast, our above study employed a significantly lower Zn^2+^ concentration, which nevertheless produced a more notable antiviral effect. To this end, here we selected rod‐shaped CNCs characterized by low cytotoxicity and high permeability for Zn^2+^ modification. By ensuring minimal cytotoxicity, we increased the plant's absorption and transport of Zn^2+^, thereby amplifying Zn^2+^‐mediated SAR. This approach lays a theoretical foundation for the informed application of Zn^2+^ in plant disease prevention and control.

## Results and discussions

### Synthesis and characterization of CNC@PDA@Zn^2+^

**Figure 1:**
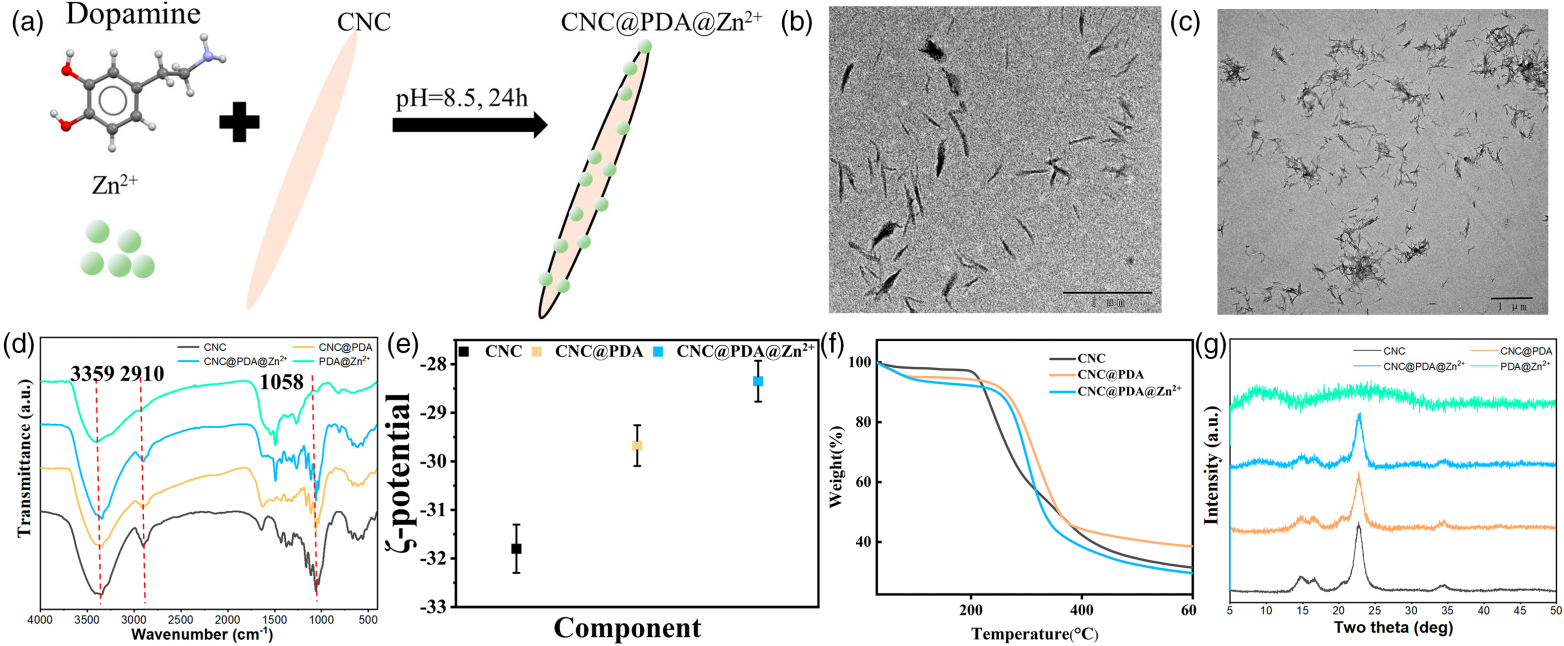
Synthesis and characterization of CNC@PDA@Zn^2+^. (a) Synthesis method of CNC@PDA@Zn^2+^. (b) The TEM image of CNC. (c) The TEM image of CNC@PDA@Zn^2+^ (d) The FTIR spectroscopy of CNC, CNC@PDA, Zn^2+^@PDA and CNC@PDA@Zn^2+^. (e) ζ‐potential of CNC, CNC@PDA, Zn^2+^@PDA and CNC@PDA@Zn^2+^. (f) TGA spectra of CNC, CNC@PDA and CNC@PDA@Zn^2+^ and (g) the X‐ray diffraction patterns obtained for CNC, CNC@PDA, Zn^2+^@PDA and CNC@PDA@Zn^2+^.

As mentioned above, although we systematically explored the minimum concentration of (CH_3_COO)_2_Zn that can induce plant disease defence, these concentrations still highly exceed the maximum Zn^2+^ tolerance of plant cells. Moreover, as the (CH_3_COO)_2_Zn concentration increases, the resistance levels significantly improve while maintaining normal growth without any high Zn^2+^ phenotypes. This phenomenon highlights the low efficiency of Zn^2+^ absorption and the high environmental residue associated with the use of (CH_3_COO)_2_Zn. A recent study has shown that plants can enhance the utilization of metal ions in complex forms (Islas‐Valdez *et al*., 2021). Therefore, we first synthesized CNC@PDA by exploiting the hydrogen bonding between CNC and PDA. This composite then served as a carrier for chelating Zn^2+^, leading to the creation of CNC@PDA@Zn^2+^ (Figure 1a). To further confirm the chemical structure of CNC@PDA@Zn^2+^, we conducted verification by Transmission Electron Microscope (TEM), Fourier‐transmitted infrared spectrometry (FTIR), Zeta potential, X‐ray diffraction (XRD) and Thermogravimetric analysis (TGA). TEM showed that after grafting Zn^2+^ on the surface of CNC, it displayed similar morphology (Figure 1b,c). In Figure 1d, the absorption peaks at 3359 cm^−1^, 2910 cm^−1^ and 1058 cm^−1^ in CNC, CNC@PDA, PDA@Zn^2+^ and CNC@ PDA@Zn^2+^ are assigned to ‐OH stretching, C–H vibration and C–O stretching vibration, respectively, which are the typical absorption peaks of CNC (Wang *et al*., 2020). Additionally, the zeta potential shifted from −31.8 ± 0.5 mV (CNC) to −29.7 ± 0.4 mV (CNC@PDA) after coating PDA. The reduced zeta potential to −27.8 ± 0.4 mV (CNC@PDA@Zn^2+^) after coordinating Zn^2+^ on the CNC@PDA surface indicates successful surface modification (Figure 1e). Additionally, TGA (Figure 1f) showed a considerable increase in the peak degradation temperature of CNC@PDA@Zn^2+^ compared with CNC, suggesting that the modification improved the thermal stability of the composite. This indicates that the PDA coating enhances the thermal resistance of the CNC@PDA@Zn^2+^ material. XRD analysis (Figure 1g) revealed that the main diffraction characteristics of CNC were still clearly present after modification, such as the diffraction peaks at 2*θ* angles around 14.7° (101), 16.6° (101¯), 22.7° (002) and 34.5° (040) (Lin *et al*., 2012). These results showed that the modification did not disturb the crystalline integrity of the main cellulose structure. Besides, it indicated the decrease in crystallinity of nanoparticles is attributed to the introduction of a dense amorphous PDA diffusion layer on the CNC structure. Table S1 presents the results of the elemental analysis of zinc, providing the anchoring of Zn^2+^ onto CNCs by inductively coupled plasma optical emission spectroscopy (ICP‐OES).

### CNC@PDA@Zn^2+^ demonstrates enhanced antiviral efficacy

**Figure 2:**
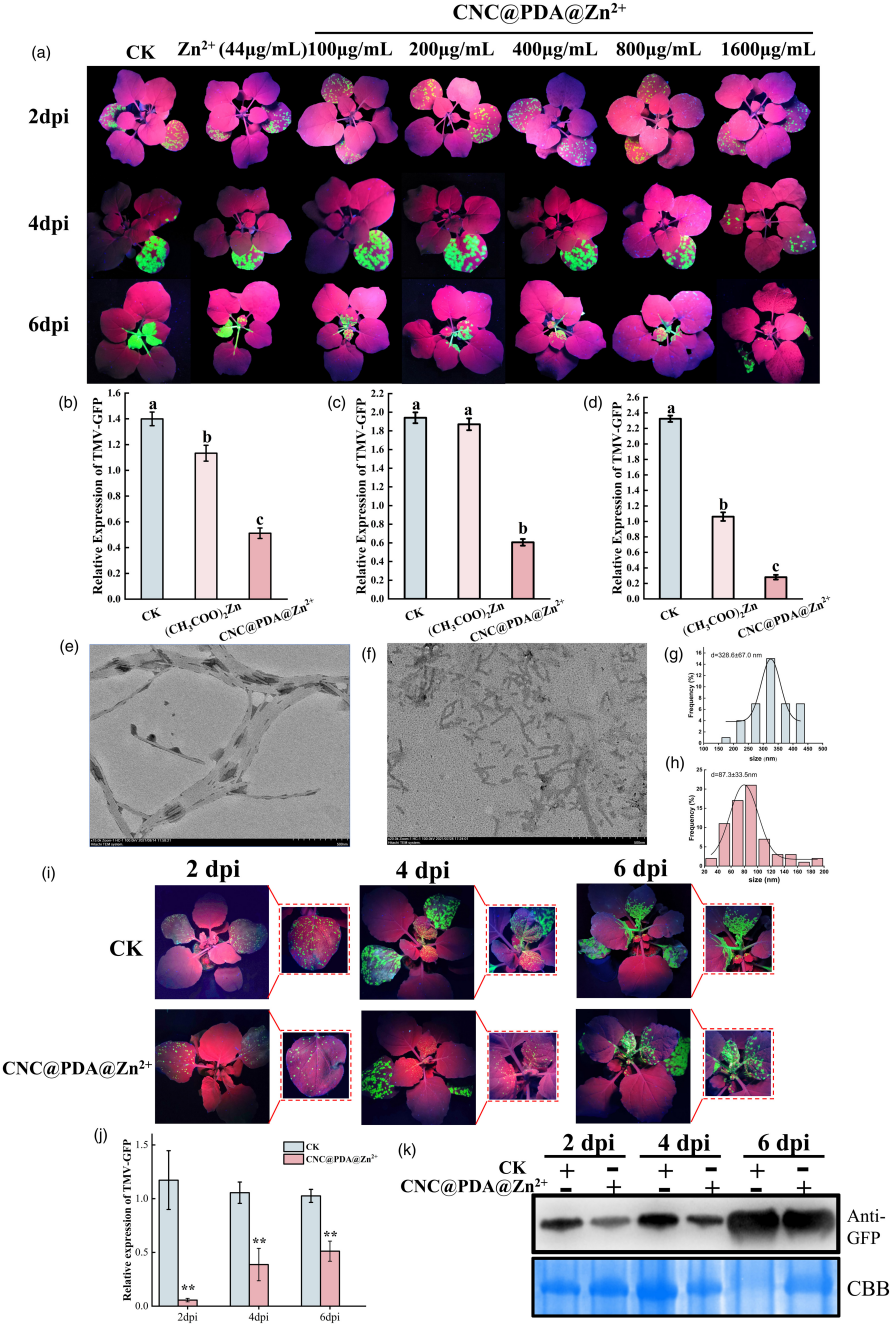
The effect of CNC@PDA@Zn^2+^ on TMV‐GFP infection *in vivo* and *in vitro*. (a) Antivirus activity of (CH3COO)_2_Zn (44 μg/mL) and CNC@PDA@Zn^2+^ (1600 μg/mL). CK as the control group, the concentration of Zn^2+^ in 44 μg/mL (CH3COO)_2_Zn was the same as the concentration of Zn^2+^ loaded in 1600 μg/mL CNC@PDA@Zn^2+^. (b–d) TMV‐GFP expression at 2 dpi, 4 dpi and 6 dpi. The accumulation of TMV‐GFP in inoculated leaves was tested at 2 dpi and 4 dpi, and the accumulation of TMV‐GFP in systemic leaves was tested at 6 dpi. (e, f) TEM images of TMV particles treated with ddH_2_O and CNC@PDA@Zn^2^+. (g, h) Distribution histograms of the length for TMV particles treated with ddH_2_O and CNC@PDA@Zn^2+^. (i) Fluorescence accumulation of TMV‐GFP at 2 and 4 days. (j) Quantitative virus accumulation at 2, 4 and 6 days by qPCR. (k) Western blot analysis of TMV‐GFP protein content. Mean values displayed in each bar followed by different letters are significantly different according to Student's *t*‐test (**p < 0.01) and LSD's multiple range test (*p* < 0.05). Vertical bars indicate standard errors (*n* = 5).

In our previous research, we observed that the antiviral activity of (CH_3_COO)_2_Zn exhibited a dose‐dependent effect within a certain range (Wang *et al*., 2022b). However, even at the lowest concentration of (CH_3_COO)_2_Zn (44 μg/mL), the Zn^2+^ concentration was approximately 237.3 μM, significantly exceeding the maximum Zn^2+^ tolerance of plant cells (Bazihizina *et al*., 2014). To address this, we utilized the dimensional properties of CNCs to reduce the usage concentration of Zn^2+^ while enhancing the overall antiviral efficacy of the material. We grafted Zn^2+^ onto a crosslinked PDA coating on rod‐shaped CNCs, resulting in CNC@PDA@Zn^2+^. Based on the ICP results (Table S1), the Zn^2+^ concentration of 1600 μg/mL CNC@PDA@Zn^2+^ is approximately equivalent to the 44 μg/mL (CH_3_COO)_2_Zn acetate solution. Subsequently, detailed experiments were conducted using a concentration of 44 μg/mL (CH_3_COO)_2_Zn as a control. Various concentrations of CNC@PDA@Zn^2+^ were sprayed daily for 3 days (10 mL per 10 plants each day). On the 4th day, GFP‐tagged TMV was inoculated onto the sixth and seventh leaves of *N. benthamiana* via rub inoculation. GFP fluorescence intensity and changes were monitored under UV light to trace TMV infection (Figure 2a). Results showed that at 2 dpi, (CH_3_COO)_2_Zn treatment exhibited some antiviral activity, whereas CNC@PDA@Zn^2+^ treatment significantly enhanced resistance, with higher concentrations correlating with increased resistance. Subsequent qPCR analysis of TMV‐GFP nucleic acid accumulation confirmed these findings (Figure 2b). By the 4 dpi, the fluorescence intensity of inoculated leaves treated with (CH_3_COO)_2_Zn and water had approached the threshold, whereas CNC@PDA@Zn^2+^ treatment resulted in slower infection progression (Figure 2c). qPCR showed that on this day, the TMV‐GFP nucleic acid levels in (CH_3_COO)_2_Zn and water‐treated leaves were similar, but significantly lower levels of TMV‐GFP were observed in CNC@PDA@Zn^2+^‐treated leaves. At 6 dpi, substantial TMV accumulation was evident in systemic leaves of water‐treated plants, significantly reduced in (CH_3_COO)_2_Zn‐treated leaves and maintained at the lowest levels in CNC@PDA@Zn^2+^‐treated leaves (Figure 2d). These results indicate that CNC@PDA@Zn^2+^ effectively delays TMV‐GFP infection. Notably, the antiviral function of CNC@PDA@Zn^2+^ exhibited a dose‐dependent response. At its maximum concentration (1600 μg/mL), the Zn^2+^ content was approximately 237.3 μM (equivalent to the Zn^2+^ content in 44 μg/mL (CH_3_COO)_2_Zn), and the material showed very strong antiviral activity, increasing efficacy more than threefold compared with the same Zn^2+^ concentration of (CH_3_COO)_2_Zn. Even when diluted 16‐fold (Zn^2+^ content approximately 16.83 μM), CNC@PDA@Zn^2+^ still exhibited excellent antiviral activity. Besides, this amount of Zn^2+^ is much lower than that in the composite nanomaterials reported previously (Cao *et al*., 2023; Zhu *et al*., 2024a). These results demonstrate that embedding Zn^2+^ in CNCs can enhance antiviral activity while reducing Zn^2+^ concentration. The significant significance of this result is that we can manipulate the reduction of Zn^2+^ content to induce stronger resistance but significantly reduce the residual Zn^2+^ in the environment.

It is well‐established that ZnO can promote the aggregation of viral particles *in vitro*, thereby inactivating the virus and ultimately inhibiting infection (Cai *et al*., 2019). However, whether CNC@PDA@Zn^2+^ possesses similar effects remains unclear. To investigate this, we first purified fresh TMV particles using differential centrifugation and then incubated them with 1600 μg/mL CNC@PDA@Zn^2+^. After 10 h, we examined the morphological changes of TMV particles under a transmission electron microscopy (TEM) microscope. Surprisingly, the treated group exhibited a significant presence of fragmented viral particles, with the average length of TMV particles being predominantly around 328.6 nm (Figure 2e,g), whereas in the CNC@PDA@ Zn^2+^‐treated group, the average length was reduced to 87.3 nm (Figure 2f,h). These findings indicate that CNC@PDA@Zn^2+^ has an *in vitro* inactivation effect on TMV particles. To confirm whether this inactivation effect alters TMV infection *in vivo*, we inoculated *N. benthamiana* leaves with the treated suspensions. At 2 dpi, the water control group exhibited similar levels of viral infection, while the CNC@PDA@Zn^2+^‐treated group showed reduced accumulation of TMV‐GFP in the inoculated leaves compared with the water control group (Figure 2i). By 4 dpi, the systemic leaves of the control groups showed a higher accumulation of TMV‐GFP compared with the CNC@PDA@Zn^2+^‐treated group (Figure 2i). The qPCR and western blot results corroborated the fluorescence observations, showing a similar trend (Figure 2j,k). These results suggest that the composite material CNC@PDA@Zn^2+^ demonstrates a strong inactivation effect on TMV‐GFP particles. These results suggest that CNC@PDA@Zn^2+^ can enhance antiviral effects by increasing *in vitro* passivation capacity while reducing Zn^2+^ content.

### CNC@PDA@Zn^2+^ enhanced Zn^2+^ uptake and transport

**Figure 3:**
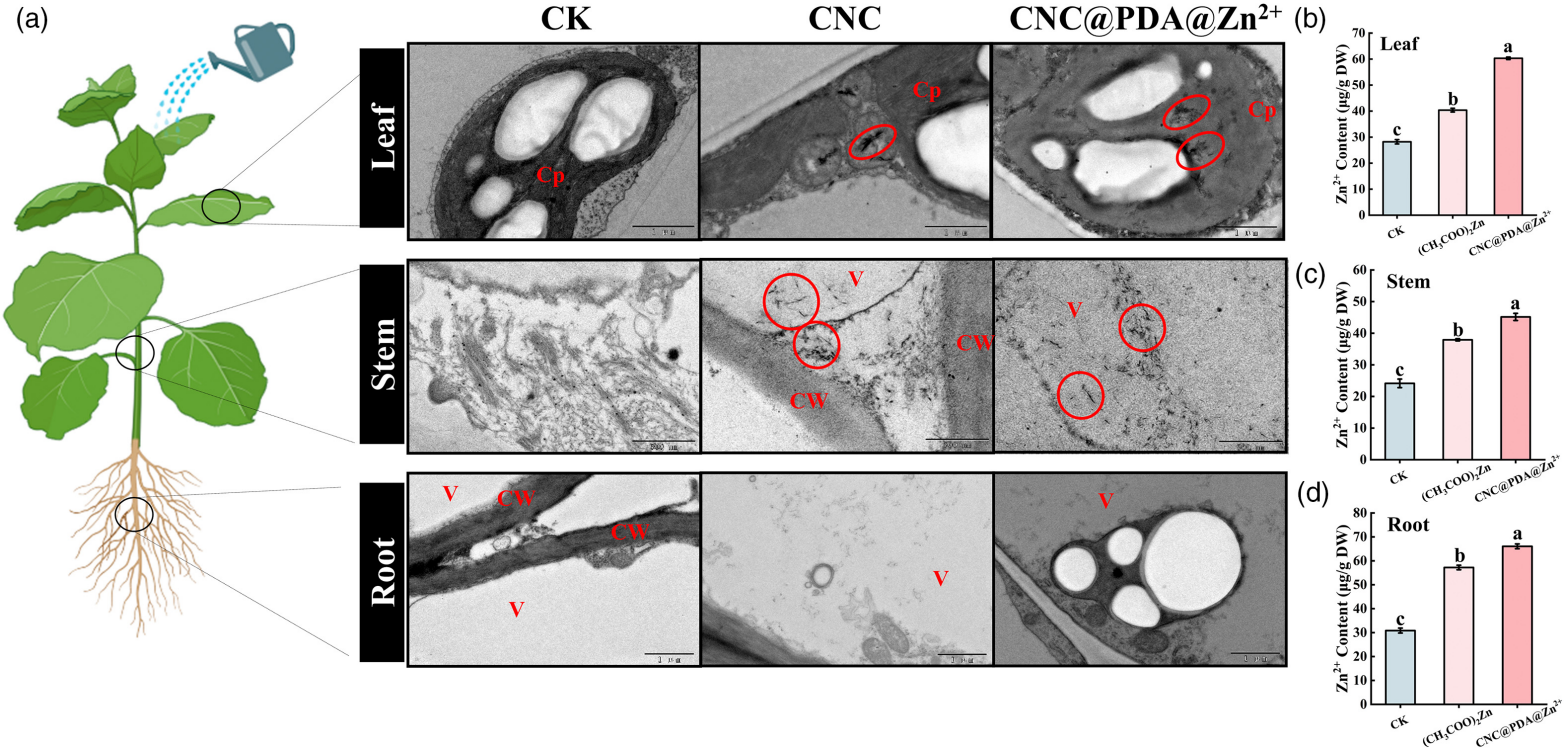
Translocation of CNC@PDA@Zn^2+^ in *N. benthamiana*. (a) TEM images of translocation in leaves, stems and roots. The red circles indicate the locations where nanomaterials are aggregated. CW—cell wall; Cp—chloroplast; V—vacuole. (b–d) Contents of Zn^2+^ in leaves, stems and roots. Mean values displayed in each bar followed by different letters are significantly different according to LSD's multiple range test (*p* < 0.05). Vertical bars indicate standard errors (*n* = 3).

The rod‐shaped CNC demonstrates low cytotoxicity and high permeability with a negatively charged surface, suggesting that the synthesized CNC@PDA@Zn^2+^ may enhance the uptake of Zn^2+^ by plant cells. To investigate this, CNC@PDA@Zn^2+^ was sprayed onto the whole *N. benthamiana* leaves for three consecutive days, and its distribution within the plant was observed using TEM after the treatments. As expected, rod‐shaped clusters were observed in the leaf tissues, indicating that CNC@PDA@Zn^2+^ could enter plant cells and aggregate within the mesophyll cells and mainly distributed in chloroplasts and the cytoplasm (Figure 3a). The content of Zn^2+^ also shows that Zn^2+^ can be loaded and entered into plant (Figure 3b). More importantly, CNC@PDA@Zn^2+^ was observed in chloroplasts (Figure 3a), demonstrating its ability to not only penetrate plant cells but also be actively transported to this key organelle. This localization suggests a targeted mechanism that may enhance its role in modulating chloroplast‐associated immune responses and antiviral defences. Besides, EDS analysis confirmed the presence of Zn^2+^ signals (Figure S1a), suggesting that Zn^2+^ remained within the nano‐carrier. In the stem tissues, dispersed CNC@PDA@Zn^2+^ structures were also observed around the vacuole, with corresponding Zn^2+^ signals detected by EDS (Figure S1b), indicating that CNC@PDA@Zn^2+^ could be transported from the leaves to the stem while still in its nano‐carrier form (Figure 3a). However, no rod‐shaped structures were observed in the elongation zone of the roots (Figure 3a). This raises the possibility that Zn^2+^ in the roots may exist in ionic form. To verify this hypothesis, we compared the Zn^2+^ content in the roots, stems and leaves after different treatments. The results showed an increased Zn^2+^ content in the roots of the treated group, with higher overall Zn^2+^ levels in CNC@PDA@Zn^2+^‐treated plants compared to those treated with (CH_3_COO)_2_Zn (Figure 3d). Notably, when measuring the Zn^2+^ content in various plant tissues following CNC@PDA@Zn^2+^ treatment, an intriguing observation emerged. After foliar application of CNC@PDA@Zn^2+^ at the highest tested concentration of 1600 μg/mL, the Zn^2+^ content in roots, stems and leaves remained relatively consistent and stable, ranging between 20 and 65 μg/g DW (Figure 3b–d). This level falls within the typical variability range of total Zn^2+^ concentrations in plant tissues, suggesting that CNC@PDA@Zn^2+^ maintains theoretical safety at this concentration. Furthermore, plant safety assessments confirmed that CNC@PDA@Zn^2+^ is non‐toxic to plants (Figure S2). Compared with water‐treated controls, CNC@PDA@Zn^2+^ treatment significantly enhanced the growth of *N. benthamiana*, as evidenced by increased plant height and dry weight. These findings underscore CNC@PDA@Zn^2+^'s dual role in promoting plant growth while ensuring safety, even at higher application rates.

Notably, rod‐shaped structures were also observed in the leaves and stems following CNC‐only treatment, suggesting that the transport of Zn^2+^ to the stem is likely facilitated by CNC. This finding leads us to hypothesize that CNC@PDA@Zn^2+^ utilizes the high permeability of CNC to transport Zn^2+^ to the stem, where Zn^2+^ is gradually released from CNC@PDA@Zn^2+^ and subsequently transferred to the roots as free Zn^2+^. However, the exact mechanism by which Zn^2+^ is efficiently regulated and transported to the roots remains unclear. To the best of our knowledge, Zn^2+^ transport within plants primarily relies on four protein families: zinc‐iron permease (ZIPs) family, responsible for transporting Zn^2+^ from the external environment or organelles into the cytoplasm (Grotz *et al*., 1998); metal tolerance proteins (MTPs) family, involved in Zn^2+^ sequestration; heavy metal ATPase (HMAs) family, which pumps Zn^2+^ into or out of organelles in an ATP‐driven manner; and yellow stripe‐like (YSLs) family, which assists in long‐distance transport and distribution of Zn^2+^ within the plant vascular system (Eren and Argüello, 2004; Huang *et al*., 2024). To elucidate how CNC@PDA@Zn^2+^ mediates long‐distance Zn^2+^ transport and efficient accumulation, we conducted a transcriptomic analysis of *N. benthamiana* leaves treated with CNC@PDA@Zn^2+^ (Figure S3 and Table S2). Compared to the water control, CNC@PDA@Zn^2+^ induced differential expression of 6040 genes; when compared to (CH_3_COO)_2_Zn, this number increased to 7575 (Figure S3a). Among these differentially expressed genes which belong to ZIPs, MTPs and HMAs family, 9 were related to Zn^2+^ transport, with the YSLs family showing no significant changes (Figure S3b). Compared to (CH_3_COO)_2_Zn, these genes exhibited an upregulation trend, suggesting that CNC@PDA@Zn^2+^ may stimulate the expression of Zn^2+^ transporters in an unknown manner, thereby enhancing Zn^2+^ transport capacity. It is important to note that the CNC@PDA@Zn^2+^ components—dopamine and cellulose—lack inherent receptors in plants. However, cellulose is recognized by receptors such as wall‐associated kinases (WAKs), leucine‐rich repeat receptor‐like kinases (LRR‐RLKs) and catharanthus roseus RLK1‐like kinases (CrRLK1L) (Man *et al*., 2020; Zhai *et al*., 2024; Zhu *et al*., 2021). The transcriptional levels of these receptors were also significantly upregulated following CNC@PDA@Zn^2+^ treatment (Figure S3c), leading us to speculate that the upregulated Zn^2+^ transport proteins might be finely regulated through phosphorylation by these cellulose‐recognizing receptors. Overall, although these transcriptomic analyses were limited to leaf tissues, they provide compelling evidence for the dual role of CNC@PDA@Zn^2+^ in regulating Zn^2+^ transport: facilitating efficient Zn^2+^ transport to the stem via the high permeability of CNC and subsequently regulating Zn^2+^ transport proteins through cellulose receptor‐mediated phosphorylation mechanisms to enhance Zn^2+^ translocation from stem to root.

### CNC@PDA@Zn^2+^ alters nsLTP2 expression and localization

**Figure 4:**
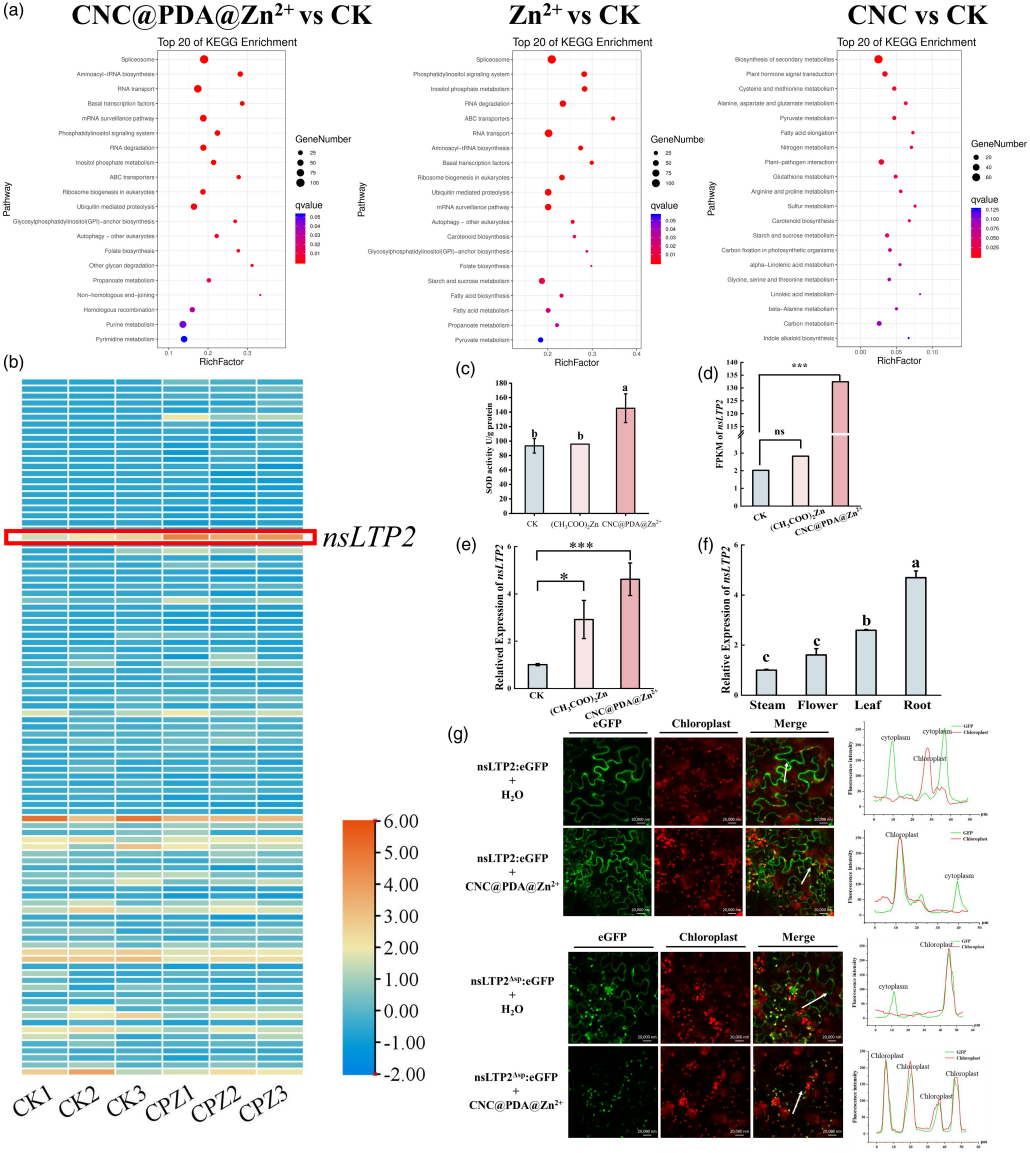
CNC@PDA@Zn^2+^ alters nsLTP2 expression and localization. (a) KEGG pathway enrichment of DEGs. (b) Heatmap representation of the expression of 100 DEGs between control and CNC@PDA @Zn^2+^. (c) SOD activity of (CH3COO)_2_Zn and CNC@PDA@Zn^2+^. (d) FPKM of *nsLTP2* in transcriptome sequence analysis. (e) qPCR validation of *nsLTP2*. (f) Expression pattern of *nsLTP2* in different tissues. (g) Subcellular localization of nsLTP2. (h) Subcellular localization of nsLTP2^Δsp^. Mean values displayed in each bar followed by different letters are significantly different according to Student's *t*‐test (*p < 0.05, **p < 0.01, ***p < 0.001) and LSD's multiple range test (*p* < 0.05). Vertical bars indicate standard errors (*n* = 3).

To investigate whether CNC@PDA@Zn^2+^ contributes to and enhances broad‐spectrum antiviral activity, we tested its inhibitory effects on the *in vivo* infection of various virus genera. The results revealed that while CNC@PDA@Zn^2+^ exhibited antiviral activity against youcai mosaic virus (YoMV), with inhibitory effects surpassing those of (CH_3_COO)_2_Zn at 5 dpi, despite its efficacy was consistently lower than its inhibitory effect against TMV (Figure S4). This disparity suggests that structural differences among these viruses may influence the direct inactivation capacity of CNC@PDA@Zn^2+^, highlighting its significant role in enhancing host resistance regulation induced by Zn^2+^. To the best of our knowledge, Zn^2+^'s known functions in plants include stabilizing enzyme structures or participating in catalytic reactions, affecting protein synthesis and gene expression, regulating chlorophyll synthesis, participating in energy metabolism and enhancing plant disease resistance (Stanton *et al*., 2022). Specifically, in terms of enhancing disease resistance, Zn^2+^ can reinforce the cell wall and increase the secretion of resistance proteins to inhibit pathogens in the apoplast, and it can also participate in hormone signalling pathways, stimulating the systemic acquired resistance (SAR) defence response (Dutta *et al*., 2024). To elucidate the role of CNC@PDA@Zn^2+^ in enhancing antiviral activity, we conducted a detailed analysis of the obtained transcriptomic profiles. The results indicated that the differentially expressed genes enriched in KEGG pathways after CNC@PDA@Zn^2+^ treatment were primarily associated with fundamental metabolic processes in plants, including spliceosome, purine metabolism, RNA transport, mRNA surveillance pathways and ubiquitin‐mediated proteolysis (Figure 4a). This suggests that CNC@PDA@Zn^2+^ treatment alters various energy and metabolic processes within the host. Additionally, we observed that CNC@PDA@Zn^2+^ induced the expression of many Zn^2+^‐binding proteins, including zinc finger proteins, zinc metalloproteases and superoxide dismutase (SOD) (Figure 4b; Dutta *et al*., 2024). Interestingly, these proteins are directly or indirectly involved in plant defence mechanisms (Dutta *et al*., 2024). For instance, the *SOD1*‐encoded enzyme activity directly influences the host's reactive oxygen species (ROS) burst, thereby regulating cell wall formation. Recent works displayed that nanoparticles loaded zinc can affect the SOD enzyme activity (Cai *et al*., 2024; Zhu *et al*., 2024a). Notably, biochemical assays further confirmed that CNC@PDA@Zn^2+^ enhances the SOD enzyme activity induced by zinc acetate within the host (Figure 4c), which is likely directly related to the increased resistance level observed.

Nevertheless, the role of CNC@PDA@Zn^2+^ in enhancing systemic acquired resistance (SAR) remains unclear. To investigate this further, we analysed the top 100 differentially expressed genes (DEGs) with the most significant expression changes and categorized these genes based on their functions, among which non‐specific lipid transfer protein (*nsLTP2*) emerged as a particularly intriguing candidate, possibly serving as a target induced by CNC@PDA@Zn^2+^ (Figure S5). Following CNC@PDA@Zn^2+^ treatment, *nsLTP2* was highly expressed, with its expression level significantly exceeding that observed in the (CH_3_COO)_2_Zn treatment group (Figure 4d,e). Lipid transfer proteins (LTPs) are small, cysteine‐rich proteins that primarily function in the transfer of various lipid molecules (Gao *et al*., 2022). They play crucial roles in plant growth, defence against pathogens and interactions with the environment (Wong *et al*., 2019). It is particularly noteworthy that Zn^2+^‐binding proteins possess highly conserved sequence motifs. In nsLTP2, we identified a C‐Xn‐C‐Xn‐CC‐Xn‐CXC‐Xn‐C‐Xn‐C motif, suggesting a potential Zn^2+^‐binding capability. However, further research is needed to validate this binding affinity. Subsequently, we examined the tissue expression pattern of *nsLTP2* in *N. benthamiana* and found that its expression was highest in the roots, followed by the leaves (Figure 4f), consistent with the expression pattern of *NbLTP1* (Zhu *et al*., 2023).

Given the importance of subcellular localization for protein function, we used bioinformatics tools to predict that nsLTP2 contains a 24‐residue N‐terminal signal peptide (SP) and is primarily localized to the cell wall. To verify these predictions, we constructed nsLTP2‐GFP fusion expression vectors driven by the cauliflower mosaic virus (CaMV) 35S promoter, as well as nsLTP2^∆SP^‐GFP fusion constructs lacking the SP, and observed their localization in cells. Experimental results showed that nsLTP2‐GFP was distributed in the cytoplasm and cell wall, while nsLTP2^∆SP^‐GFP was found in the cytoplasm and chloroplasts. Interestingly, when CNC@PDA@Zn^2+^ was applied to cells expressing these fusion proteins, their localization underwent significant changes. For nsLTP2‐GFP, intracellular protein accumulation decreased and concentrated some in the chloroplast, with more protein accumulating extracellularly (Figure 4g). By contrast, nsLTP2^∆SP^‐GFP became more concentrated in the chloroplasts, with the Pearson correlation curve of the GFP channel and chloroplast fluorescence channel completely overlapping (Figure 4h). Additionally, western blot analysis confirmed that CNC@PDA@Zn^2+^ treatment not only altered nsLTP2/nsLTP2^∆SP^ localization but also increased nsLTP2 protein accumulation (Figure S6). This result strengthens the correlation between CNC@PDA@Zn^2+^ and nsLTP2.

Extensive research on alterations in protein localization indicates a critical role in plant stress resistance. It was found that knockout protein‐only RNase P 2 (PRORP2) which contains a conserved nuclear localization signal (NLS) can change the subcellular localization and exhibited significantly enhanced resistance to oilseed rape mosaic virus (ORMV) and turnip yellow mosaic virus (TYMV) (Gobert *et al*., 2021). Changes in protein localization are often accompanied by post‐translational modifications (PTMs). Huang *et al*. (2021) reported that the lipid transfer proteins DgTIL1 and DgnsLTP interact and that lysine crotonylation enhances their interaction, preventing DgnsLTP degradation. This stabilization further promoted peroxidase (POD) activity and improved cold tolerance in plants. Medina‐Puche *et al*. (2020) found that a virus‐encoded protein, upon activation of plant defence responses, relocalized from the plasma membrane to the chloroplast. This chloroplast targeting suppressed chloroplast‐dependent SA biosynthesis and required both an N‐myristoylation site and a chloroplast transit peptide. The G‐type nsLTP possesses a glycosylphosphatidylinositol (GPI) anchor signal at its C‐terminus, allowing membrane anchoring through GPI modification. The protein can then be released by phosphatidylinositol‐specific phospholipase C, anchoring it on or near the plasma membrane, where it may be involved in callose deposition and intercellular communication (Edstam *et al*., 2014; Simpson *et al*., 2009).

Previous studies have demonstrated that nsLTPs can bind palmitic acid via their hydrophobic cavity, thereby facilitating intracellular trafficking (Salminen *et al*., 2016). S‐palmitoylation, a lipid modification, involves the addition of a C16 palmitoyl group from palmitoyl‐CoA to the thiol side chain of cysteine residues by palmitoyl acyltransferases (PATs). This modification introduces a long hydrophobic chain that anchors the protein to cellular membranes. Notably, this membrane association is reversible, as thioesterases can cleave the thioester bond, allowing palmitoylation to function as a dynamic switch for membrane targeting. Protein palmitoylation has been shown to regulate the activity of the immune receptor P2K1 by preventing its phosphorylation and degradation, thereby modulating extracellular ATP (eATP)‐induced immune signalling in plants (Chen *et al*., 2021). Interestingly, nsLTP2 contains a hydrophobic cavity and a putative PAT‐targeting site exposed after the signal peptide (C28 and G29 were identified as the modification site), suggesting its potential involvement in lipid trafficking and subcellular localization via cargo‐mediated lipid modification. Moreover, a canonical N‐myristoylation site is located adjacent to the PAT modification site—two lipid modifications that often act in concert. Additionally, the subcellular localization of nsLTP2^Δsp^ was altered in the presence of CNC@PDA@Zn^2+^, indicating that CNC@PDA@Zn^2+^ may modulate the acylation‐dependent localization of nsLTP2^Δsp^.

### nsLTP2 as a positive regulator induces plant resistance

**Figure 5:**
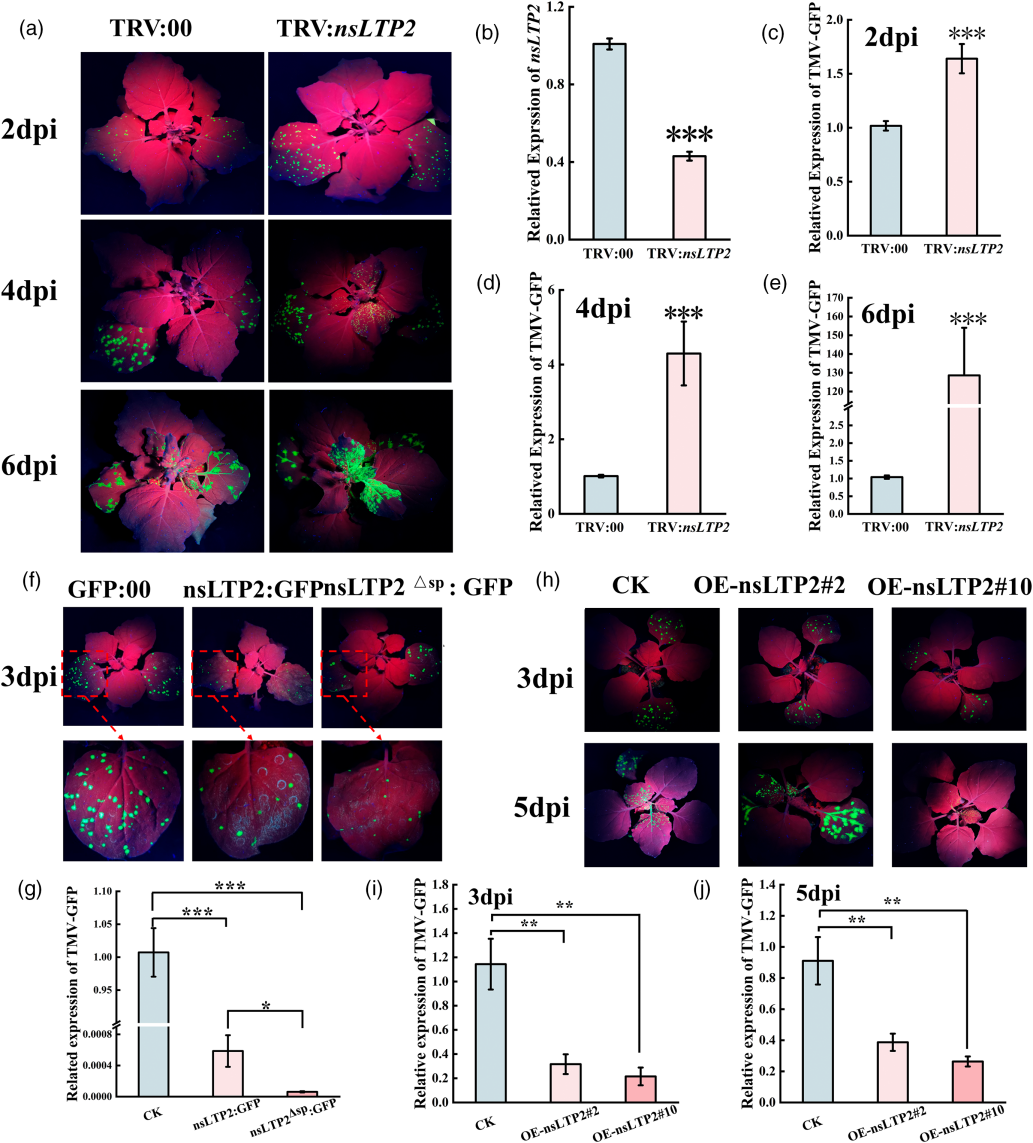
nsLTP2 as a positive regulator induces plant resistance. (a) Symptom of TMV‐GFP infection after silencing *nsLTP2* for 12 days. (b) The expression of *nsLTP2* on silenced plants. (c–e) Accumulation of TMV‐GFP at 2, 4 and 6 dpi was detected by RT‐qPCR. TMV‐GFP in the inoculated leaves was quantified at 2 dpi. 4 and 6 dpi were quantified the TMV‐GFP in the systemic leaves. (f–h) Symptom maps of OE‐nsLTP^Δsp^ and WT plants inoculated with TMV‐GFP; (i, j) TMV RNA levels in OE‐nsLTP^Δsp^ and WT plants were detected by qPCR at 3 and 5 dpi. Mean values displayed in each bar followed by different letters are significantly different according to Student's *t*‐test (*p < 0.05, **p < 0.01, ***p < 0.001). Vertical bars indicate standard errors (*n* = 3).

To understand the functional significance of CNC@PDA@Zn^2+^‐induced changes in nsLTP2 localization in plant resistance, we tested the antiviral function of nsLTP2. While the role of NbLTP1 in TMV infection is well characterized, we systematically explored the role of nsLTP2 during TMV infection. Stress expression analysis revealed that *nsLTP2* expression significantly increased in inoculated leaves at 2 dpi with TMV‐GFP, and its expression also markedly elevated in systemic leaves at 6 dpi (Figure S7), suggesting a potential role for nsLTP2 in antiviral defence. Next, we utilized tobacco rattle virus (TRV)‐mediated gene silencing to analyse nsLTP2's role in TMV resistance. Following the agroinfiltration of *N. benthamiana* leaves with TRV1 + TRV2 (TRV:00) or TRV1 + TRV2:nsLTP2 (TRV:nsLTP2) for 12 days, RT‐qPCR analysis revealed that *nsLTP2* expression in systemic leaves was significantly lower in TRV:nsLTP2 plants compared with controls (Figure 5b). We then mechanically inoculated the 6th and 7th leaves with TMV‐GFP and observed the movement of GFP under UV light at 2, 4 and 6 dpi to track TMV distribution. As shown in Figure 5a, GFP fluorescence signals were observed in the inoculated leaves at 2 dpi, and qPCR analysis demonstrated that TMV‐GFP nucleic acid levels were significantly higher in silenced plants than in controls (Figure 5c). At 4 dpi, GFP signals appeared in the systemic leaves of silenced plants, whereas no GFP signals were detected in the systemic leaves of control plants; qPCR analysis of viral nucleic acids yielded similar results (Figure 5d). By 6 dpi, the GFP signal was more pronounced in silenced plants, and TMV‐GFP accumulated significantly in their systemic leaves (Figure 5e). These results indicate that *nsLTP2* silencing facilitates TMV‐GFP infection. Subsequently, we compared the disease resistance conferred by nsLTP2 and nsLTP2^ΔSP^. We fused nsLTP2 and nsLTP2^ΔSP^ with eGFP via a P2A linker, using eGFP:00 as a control. Following transient expression and TMV‐GFP inoculation, we found that by 3 dpi, the control leaves contained numerous GFP fluorescence spots, while the treated leaves had significantly fewer spots, with the lowest number observed in the nsLTP2^ΔSP^ overexpression group (Figure 5f). qPCR results also showed that TMV‐GFP nucleic acid levels were significantly lower in the nsLTP2 and nsLTP2^ΔSP^ overexpression groups compared with the control, with the nsLTP2^ΔSP^ group containing the least amount of TMV‐GFP nucleic acids (Figure 5g). We then provided more compelling genetic evidence by stably overexpressing nsLTP2^ΔSP^ under the CaMV 35S promoter and analysing its detailed antiviral phenotype. Consistent with the transient expression results, two independent overexpression lines exhibited remarkable antiviral activity, especially line #10 (Figure 5h–j). These findings suggest that chloroplast‐localized nsLTP2^ΔSP^ confers stronger antiviral functions. As lipid transfer proteins, LTPs maintain the stability of organelle membranes by transporting lipids between various organelles (Gao *et al*., 2022). For nsLTP2 containing the SP, CNC@PDA@Zn^2+^ promotes its secretion to the extracellular space, potentially supporting the hypothesis that it stabilizes cell wall structures by transporting lipids (Gao *et al*., 2022). However, we believe that its more crucial role lies in executing PR14 functions. LTP2 may have potential PR14‐related functions, and the upregulation of this pathogenesis‐related protein directly contributes to host resistance (Gao *et al*., 2022). Nevertheless, nsLTP2^ΔSP^ demonstrates stronger resistance and accumulates in chloroplasts under CNC@PDA@Zn^2+^ treatment. Chloroplasts are a primary battleground in plant–virus interactions, with chloroplast homeostasis directly influencing viral symptom development, and several chloroplast proteins exhibit antiviral activity (Liu *et al*., 2021; Zhao *et al*., 2016). Notably, CNC@PDA@Zn^2+^ seems to be able to enter chloroplasts and enhance the expression of various chloroplast‐associated proteins (Figure S8), strongly suggesting that CNC@PDA@Zn^2+^‐induced nsLTP2 stabilizes chloroplast membrane structures by transporting lipids, leading to enhanced antiviral defence. Furthermore, we cannot entirely dismiss an important yet easily overlooked possibility: In roots lacking chloroplasts, nsLTP2 may function as a PR14 resistance protein when induced by CNC@PDA@Zn^2+^, while in chloroplast‐rich leaves, nsLTP2 suppresses viral infection by maintaining chloroplast homeostasis. However, these hypotheses require further evidence for confirmation.

Furthermore, Zn^2+^ is known to facilitate cell wall fortification and is essential for chlorophyll synthesis (Dutta *et al*., 2024; Stanton *et al*., 2022), making it difficult not to associate these processes with the function of nsLTP2. We hypothesize that nsLTP2 may bind Zn^2+^ and subsequently transport lipids to the cell wall and chloroplasts. This lipid‐mediated process could involve the reversible transfer of Zn^2+^ to the cell wall and chloroplasts, where it plays a crucial role. Notably, CNC@PDA@Zn^2+^ appears to accelerate this process, possibly due to the increased intracellular Zn^2+^ levels introduced by CNC@PDA@Zn^2+^ or a chain reaction triggered by the CNC receptor hypothesis. However, more detailed genetic evidence is required to confirm these mechanisms. Regardless, these results support the role of CNC@PDA@Zn^2+^ in enhancing resistance by inducing nsLTP2.

### The induction of nsLTP2 by CNC@PDA@Zn^2+^ is dependent on the SA pathway for its disease response

**Figure 6:**
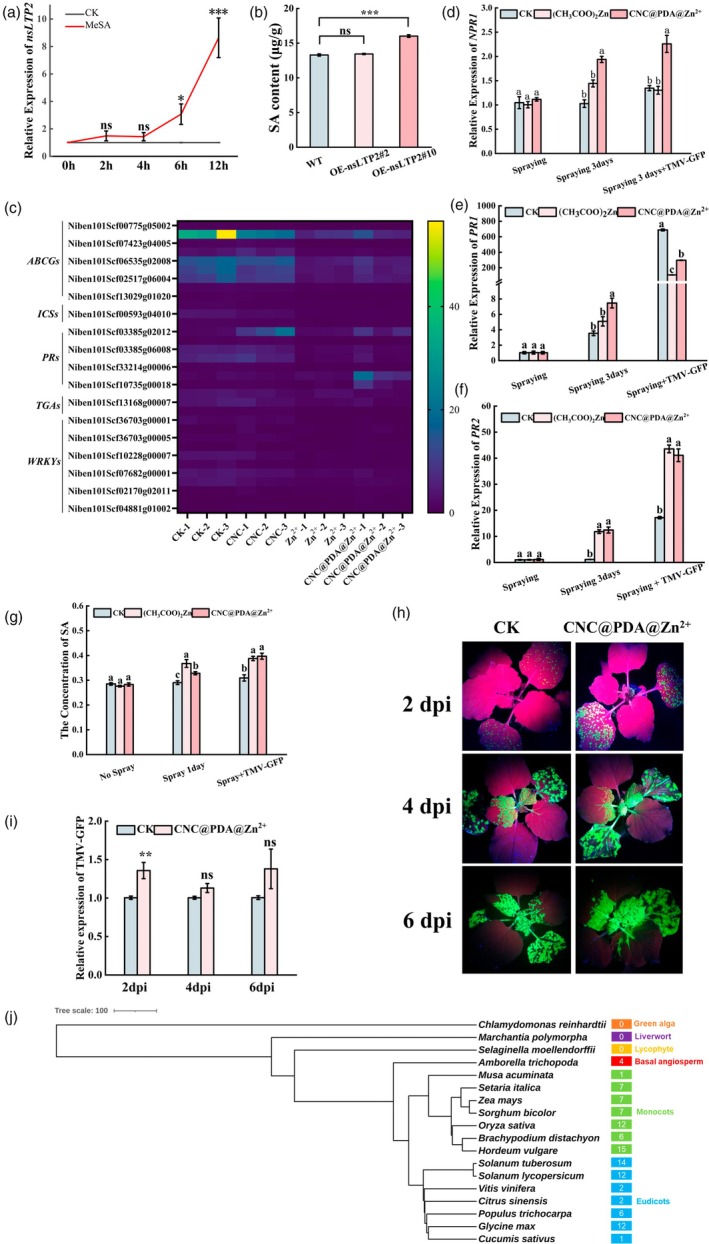
Conserved nsLTP2 is involved in the SA‐mediated resistance pathway. (a) Relative expression of *nsLTP2* after being treated with MeSA. (b) SA content in two nsLTP overexpression lines. (c) DEGs related to SA. (d–f) Response of *NPR1*, *PR1* and *PR2* after spraying CNC@PDA@Zn^2+^ 1 h, spraying CNC@PDA@Zn^2+^ 3 days and spraying CNC@PDA@Zn^2+^ 3 days then inoculate TMV‐GFP. (g) SA content after CNC@PDA@Zn^2+^ treatment. (h) Accumulation of TMV‐GFP after spraying CNC@PDA@Zn^2+^ in *NahG* at 2 dpi (inoculated leaves) and 6 dpi (systemic leaves). (i) Related expression level of TMV‐GFP in *NahG* after treated with CNC@PDA@Zn^2+^. (j) Evolutionary relationships of nsLTP2. Mean values displayed in each bar followed by different letters are significantly different according to Student's *t*‐test (*p < 0.05, **p < 0.01, ***p < 0.001) and LSD's multiple range test (*p* < 0.05). Vertical bars indicate standard errors (*n* = 3).

The SAR in plants is intrinsically linked to SA, and Zn^2+^ plays a crucial role in this process as well. Previous studies have already associated Zn^2+^ with SAR (Jung *et al*., 2009; Nadia *et al*., 2018), but whether Zn^2+^ can promote SA accumulation by regulating the expression of nsLTP2 remains unclear. Notably, whether it functions as a PR14 protein or stabilizes chloroplast membrane structures, nsLTP2 appears to be closely linked to SA, as PR proteins exert their functions through the SA synthesized in chloroplasts. To explore this, we first treated *N. benthamiana* leaves with the SA analog MeSA and quantified the changes in *nsLTP2* expression during this process. The results showed that *nsLTP2* expression increased over time following MeSA treatment, indicating that *nsLTP2* is an SA‐inducible gene (Figure 6a). We then measured the SA content in two stable overexpression lines of nsLTP2^ΔSP^ and found that both lines exhibited increased SA levels, even though there is no significant difference between OE‐nsLTP2#2 and WT, but the expression level of *nsLTP2* in OE‐nsLTP2#2 is lower than OE‐nsLTP2#10, which suggested the dose effect between *nsLTP2* and SA content, which suggests a potential link between nsLTP2 and SA (Figure 6b). Additionally, we searched the transcriptome database for SA‐related genes induced by CNC@PDA@Zn^2+^ and found that CNC@PDA@Zn^2+^ enhanced the expression levels of several SA‐related genes, including *nsLTP2* (Figure 6c). We further quantified the expression of key genes involved in SA synthesis and response, such as *NPR1*, pathogenesis‐related gene 1 (*PR1*) and pathogenesis‐related gene 2 (*PR2*). The results showed that both CNC@PDA@Zn^2+^ and (CH_3_COO)_2_Zn treatments upregulated the expression of *NPR1*, *PR1* and *PR2*, with CNC@PDA@Zn^2+^ promoting higher expression levels of *NPR1* and *PR1* (Figure 6d–f). Subsequently, we inoculated TMV‐GFP after treating plants with CNC@PDA@Zn^2+^ and (CH_3_COO)_2_Zn to examine the expression of SA‐related genes under these complex conditions. The results demonstrated that under TMV‐GFP infection, both (CH_3_COO)_2_Zn and CNC@PDA@Zn^2+^ treatments induced high expression of *PR2* (Figure 6f), while CNC@PDA@Zn^2+^ also promoted *NPR1* expression (Figure 6d). Furthermore, SA levels increased following (CH_3_COO)_2_Zn and CNC@PDA@Zn^2+^ treatments (Figure 6g). Notably, after TMV‐GFP inoculation, the SA content in the CNC@PDA@Zn^2+^‐treated group was slightly higher than in the (CH_3_COO)_2_Zn‐treated group (Figure 6g). These results suggest that the resistance induced by CNC@PDA@Zn^2+^ in TMV‐GFP‐infected plants is more stable. To determine whether the resistance induced by CNC@PDA@Zn^2+^ in *N. benthamiana* is entirely dependent on the SA pathway, we treated transgenic *NahG* plants, which express salicylate hydroxylase and are completely deficient in SA, with CNC@PDA@Zn^2+^ and observed whether their antiviral phenotype changed. The results showed that at 2 dpi, *NahG* plants displayed stronger fluorescence signals in their leaves compared with the control, but this difference significantly diminished at 4 and 6 dpi (Figure 6h). RT‐qPCR analysis indicated that the inhibitory effect of CNC@PDA@Zn^2+^ on TMV‐GFP infection was completely lost at 4 and 6 dpi (Figure 6i). These findings highlight the crucial role of CNC@PDA@Zn^2+^ in inducing host systemic resistance, and this SAR resistance is largely dependent on SA signalling.

### Evolutionary conservation of nsLTP2 in flowering plants

Finally, we conducted an in‐depth analysis of the evolutionary relationships of nsLTP2 across the plant lineage, given its potential relevance to the functional scope of CNC@PDA@Zn^2+^. Notably, nsLTP2 originated in angiosperms, as it was first identified in *Amborella trichopoda*. *A. trichopoda* is the sole surviving representative of the basal sister lineage of all extant angiosperms, suggesting that nsLTP2 is one of the earliest genes to have emerged during the evolution of angiosperms. This highlights its critical role in the origin and early evolution of flowering plants. Subsequently, these nsLTP2 genes underwent duplication across different species, leading to a significant number of homologues in key crops such as *Solanum lycopersicum* (12 copies), *Oryza sativa* (12 copies) and *Zea mays* (7 copies) (Figure 6j). These findings suggest that CNC@PDA@Zn^2+^ may broadly induce the expression of nsLTP2 across this vast phylogenetic group of angiosperms and that there are multiple nsLTP2 targets in some key crops. This further indicates the significant role of CNC@PDA@Zn^2+^ in inducing resistance defences throughout the plant kingdom.

**Figure 7:**
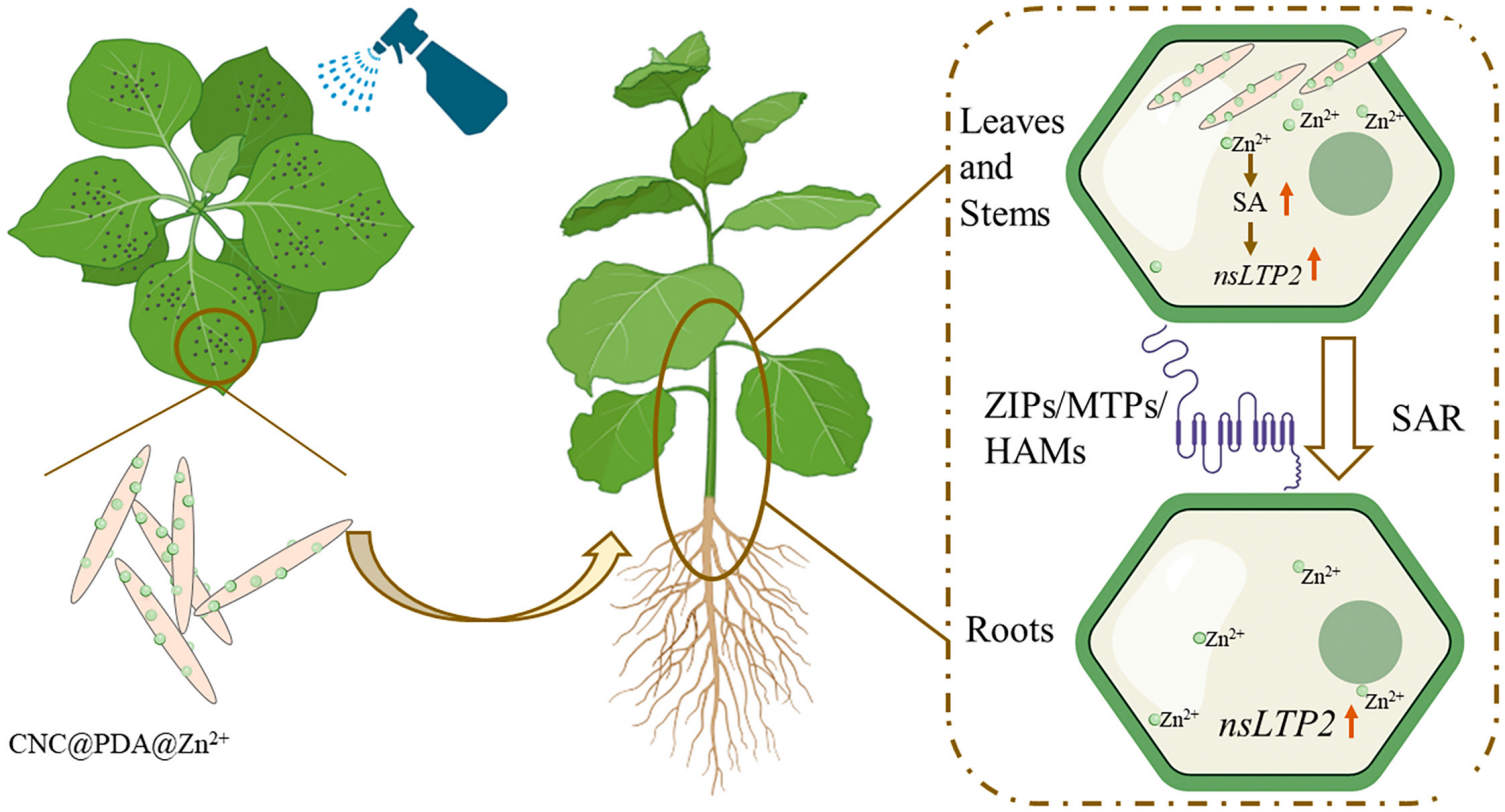
Schematic representation of CNC@PDA@Zn^2+^‐induced Zn^2+^ transport and its activity of induced plant immunity.

In summary, we developed a rode‐shape nanomaterial, CNC@PDA@Zn^2+^, by embedding Zn^2+^ into a PDA coating on CNCs. This innovative material demonstrated enhanced efficiency in controlling plant virus infection and facilitating trace element transport within plants, while significantly reducing Zn^2+^ usage, positioning it as a key player in sustainable agricultural practices. Mechanistic analyses revealed that CNC@PDA@Zn^2+^ induced a SA‐mediated defence response by upregulating the secretion and expression of nsLTP2 across different organelles (Figure 7). Notably, this mechanism appears to be highly conserved among angiosperms. Our study presents a novel strategy for efficient Zn^2+^ delivery in plants, strengthening plant immune responses and resistance to pathogens, thereby advancing the sustainable use of Zn^2+^ as a plant disease control agent.

## Materials and methods

### Plant materials and growth conditions

*Nicotiana benthamiana* seeds were sterilized with 75% alcohol and inoculated in MS medium. After 1 week, they were transferred into soil. The growing condition was under a 16 h day/8 h night cycle, 26 °C and 80% relative humidity. The plants were cultured to the six‐leaves to eight‐leaves stage for the next experiment, and fresh leaves were collected and frozen in liquid nitrogen for subsequent analysis.

### Synthesis of CNC@PDA@Zn^2+^

The CNCs were prepared by hydrolyzing cotton linter with sulfuric acid according to our previous work (Shen *et al*., 2021). The purified CNCs were used to prepare CNC@PDA@Zn^2+^. Dopamine hydrochloride (DA·HCl, 98%) and zinc chloride (ZnCl_2_) were purchased from Shanghai Aladdin Biochemical Technology Co. Ltd. (Shanghai). Trimethylaminomethane (Tris) was purchased from Shanghai Yuanye Bio‐Technology Co. Ltd. (Shanghai). Sodium hydroxide (NaOH, 96.0%) and hydrochloric acid (HCl, 36%–38%) were purchased from Chongqing Chuandong Chemical Co. Ltd. (Chongqing).

### Characterization of CNC@PDA@Zn^2+^

The FTIR spectra of CNC, CNC@PDA, CNC@PDA@Zn^2+^ and PDA@Zn^2+^ were characterized by a Nicolet 170SX Fourier Transform (Madison, WI, USA) with anhydrous KBr in the range of 4000–500 cm^−1^ at an attenuated total reflection cell by averaging 32 spectra with a resolution of 4 cm^−1^. Zn^2+^ content was measured by inductively coupled plasma atomic emission spectroscopy (ICP‐AES) (iCAP7000, American). The morphology of CNC and CNC@PDA@Zn^2+^ was measured by transmission electron microscope (TEM) (Talos F200X). The ζ‐potential of CNC, CNC@PDA and CNC@PDA@Zn^2+^ was measured by a Zetasizer nano ZS (Malvern, UK). X‐ray diffraction (XRD, D8 ADVANCE, Bruker) was employed to investigate the interaction of CNCs with PDA@Zn^2+^. Thermogravimetric analysis (TGA, STA 499 F5/F3 Jupiter, NETZSCH) was conducted to characterize the thermal stability and decomposition behaviour of composites.

### Virus inoculation

For TMV‐GFP inoculation, the friction inoculation method was employed to eliminate potential experimental interference from *Agrobacterium*, which is commonly associated with the *Agrobacterium* infiltration method. Specifically, 0.1 g of fully TMV‐GFP‐infected *N. benthamiana* leaves were collected and ground into a fine powder using a mortar and pestle under liquid nitrogen. The resulting powder was dissolved in PBS buffer (pH = 7.4), and the suspension was adjusted to an OD_600_ of 1. The prepared solution was then used to inoculate designated leaves via friction inoculation.

### Western blot

For protein extraction, 0.1 g of leaf tissue was ground into a fine powder in a pre‐chilled mortar with liquid nitrogen. Protein lysis buffer (0.1 M Tris–HCl, pH 7.4, 1% SDS, 0.02% β‐mercaptoethanol) was added to the powder, and the mixture was incubated on ice for 30 min. The lysate was centrifuged at 5000 *g* for 15 min at 4 °C, and the resulting supernatant was collected. SDS‐protein loading buffer was added to the supernatant, and the mixture was boiled at 95 °C for 5 min to denature the proteins. The prepared protein samples were subjected to SDS‐PAGE using a Mini‐PROTEAN^®^ Tetra Cell system and subsequently transferred onto a PVDF membrane for western blot analysis. The membrane was probed with a mouse anti‐GFP monoclonal antibody (ABclonal, AE012) as the primary antibody and HRP‐conjugated goat anti‐mouse IgG (H + L) (ABclonal, AS003) as the secondary antibody.

### RNA extraction and cDNA synthesis

RNA was extracted using an Eastep^®^ Super Total RNA Extraction Kit (Promega, LS1040, Beijing, China). The RNA sample was then reverse transcribed with a PrimeScript™ RT Reagent Kit (TaKaRa, RR037A, Shiga, Japan) in a 10 μL reaction.

### RNA‐seq

The *N. benthamiana* leaves of CNC@PDA@Zn^2+^ treatments (PZ‐1, PZ‐2 and PZ‐3), (CH_3_COO)_2_Zn treatments (Z‐1, Z‐2 and Z‐3), CNC treatments (CNC‐1, CNC‐2 and CNC‐3) and water treatments (CK‐1, CK‐2 CK‐3) were collected, and RNA was extracted using RNAiso Plus reagent (Takara, Japan). mRNA was isolated with oligo (dT) cellulose and broken into short fragments (200 nt) by adding a fragmentation buffer. First‐strand cDNA was generated using random hexamer‐primed reverse transcription, and second‐strand cDNA was synthesized by DNA polymerase I and RNase H. After that, the synthesized cDNA fragments were purified and subjected to end pairing by adding a single ‘A’ base, and ligated with Illumina adapters. The ligation products were size‐fractionated by agarose gel electrophoresis, and fragments were excised for PCR amplification. The amplified fragments were sequenced using Illumina HiSeqTM 2500 by Gene Denovo Co. (Guangzhou, China). De novo transcriptome assembly and differentially expressed gene (DEGs) analysis were described. In brief, the raw reads obtained by the sequencing platform were subjected to quality control (QC) and filtered to obtain high‐quality clean reads. After that, clean reads were aligned to the reference genome (Niben.genome.v1.0.1). After performing the QC, a follow‐up analysis was performed, followed by quantitative analysis and structural analysis. Quantitative analysis includes quantification of genes, exons and transcripts. Subsequent differential expression analysis was based on quantitative analysis, functional enrichment analysis and time‐series analysis. Structural analysis consists of alternative splicing analysis, gene structure optimization, new transcript analysis, SNP/InDel analysis and gene fusion analysis.

### Real‐time quantitative PCR

For real‐time quantitative PCR (qPCR), qTOWER2.0 real‐time PCR (Analytikjena, Germany) and a QuantiNova™ SYBR Green PCR Kit (QIAGEN, Germany) were used to determine the relative expression levels of target genes. Three biological replicates were performed for each sample. *ACTIN* was selected as an internal control. Quantification of the relative changes in gene transcript levels was performed using the 2^−▵▵Ct^ method. All primers are shown in Table S3.

### Virus particle purification and TEM observation

To purify the virus particles, the first step is to inoculate TMV and collect 4 g leaves after 4 days. Add 0.2 mol/L PBS buffer (pH = 7.2) mixed with 1% β‐mercaptoethanol (99 mL PBS + 1 mL β‐mercaptoethanol) and ground it into a homogenate (1 g tissue per 1 mL buffer). The filter collects the supernatant as the crude extract. Under magnetic stirring, add 8% n‐butanol (8 mL per 100 mL of solution), continue stirring for 15 min and then centrifuge at 9279 *g* for 20 min. Collect the supernatant, add 4% NaCl and 4% PEG6000 (4 g each per 100 mL solution), stir for 1.5 h and centrifuge at 9279 *g* for 15 min. Collect the precipitate, resuspend it in 0.01 mol/L PBS buffer (pH 7.2) and centrifuge at 5939 *g* for 5 min. Collect the precipitate, resuspend it in 0.01 mol/L PBS buffer (pH 7.2) and centrifuge at 5939 *g* for 5 min. The supernatant is the purified TMV extract, which is stored at 4 °C.

Mix the purified TMV particles with nanomaterials and ddH_2_O as the control. Allow all mixtures to interact *in vitro* at 25 °C for 3 h. Observe the TMV morphology under a JEOL JEM‐2100 electron microscope. Eight fields of view were analysed for each treatment, and representative TEM images were selected. The control group was processed using the same procedure.

For the CNC nanomaterial transport in the plant, the leaf samples were sampled after spraying 3 days, stem samples were sampled at 4 days after spraying 3 days and the root samples were sampled at 7 days after spraying 3 days.

### Vector construction

For expression vector construction, two segments encoding *nsLTP2* (Niben101Scf07951g02011) and *nsLTP2* ^*▵SP*^ (without the signal peptide) were designed with *Xba*I restriction sites and a 20‐bp sequence overlapping with the region surrounding *Xba*I in the pART27‐eGFP vector. These fragments were seamlessly cloned into the pART27‐eGFP backbone, resulting in the binary expression vectors pART27‐nsLTP2‐eGFP and pART27‐nsLTP2^▵SP^‐eGFP. Similarly, the pART27‐nsLTP2^▵SP^‐7 × Myc binary expression vector was constructed using the same seamless cloning approach. To construct the silencing vector, a 200‐bp optimal silencing fragment for *nsLTP2* was identified using the VIGS tool (https://vigs.solgenomics.net/). This fragment was inserted into the pTRV2 vector at the *Xba*I and *Xho*I sites to generate the pTRV2‐nsLTP2 vector. All primers are shown in Table S3.

### *N. benthamiana* leaves infiltration and confocal observation

Transient expression was performed as previously described, with minor modifications. Briefly, the constructs pART27‐nsLTP1‐GFP, pART27‐nsLTP2^▵SP^‐eGFP, pTRV1, pTRV2‐nsLTP2 and P19 were transformed into *Agrobacterium tumefaciens* strain GV3101. The *Agrobacterium* cultures were activated with acetosyringone and prepared for infiltration. For expression vectors (pART27‐based constructs), the cultures were adjusted to an OD_600_ of 0.5. For silencing vectors (pTRV‐based constructs), the cultures were adjusted to an OD_600_ of 0.3 and co‐infiltrated with P19 (OD_600_ = 0.3) to enhance expression. The mixtures were infiltrated into the leaves of *N. benthamiana* using a syringe without a needle.

For confocal observation, *N. benthamiana* leaves at the 6‐leaf stage were infiltrated and leaf discs were isolated from the infiltrated leaves and visualized using a LSM780 confocal laser scanning microscope equipped with a 40*/1.2 water‐immersion objective (Zeiss, Germany). GFP‐derived fluorescence was excited a 488 nm, and emission was captured with a 505‐ to 530‐nm filter.

For generating a Pearson‐correlated curve, we use ImageJ and split the image into green and red colour channels, then select the same place but in different channels, choose plot profile to analyse the Pearson's correlation coefficient, save the data as txt and import the data into Excel to have the figure.

### SOD activity detection

The enzyme activity was determined according to the previous report (Lv *et al*., 2020). PBS was used to extract total protein from the treated *N. benthamiana* leaves, and enzyme activity kits were used to measure the activities SOD (at 560 nm) (Sinobest, YX‐C‐A500) according to the manuals, with three repetitions for each treatment.

### Detection of SA in leaves

For SA quantification, 50 mg leaf tissue was finely ground in liquid nitrogen and extracted with 2 mL pre‐cooled 80% methanol, then sealed with plastic wrap and cold‐soaked at 4 °C overnight. Centrifuge at 4 °C, 5000 r/min for 10 min, take the supernatant and the residue continue to extract with 80% methanol. The supernatant was pooled by sonication two times, and the aqueous phase added 2 mL petroleum ether to decolorize 3 times, extract the aqueous phase with ethyl acetate 3 times, then blow it and add an acetic acid solution (pH = 3.5), purify through C18 column (Agilent C18,250*4.6 mm; 5 μm), elution with methanol, collect the eluate and dry before dissolving with mobile phase to a constant volume of 1 mL, shake and mix the liquid then pass through a 0.22 μm filter membrane and be tested. The quantification of SA was determined by HPLC (Agilent 1200), the equipment settings according to previous work (Cho *et al*., 2013). Three independent replicates were performed, with each experiment containing three biological repeats.

### Elemental analyses

The concentration of Zn and Si in the tobacco plants (leaf, stem and root) was assessed via inductively coupled plasma mass spectrometry (ICP‐MS; ELAN DRC II, Perkin Elmer Inc.). Specifically, dried plant samples were fully digested in 6 mL of HNO_3_ and hydrogen peroxide (5:1) solution at 150 °C. Then, deionized water was added to the solution until the volume reached 50 mL. The supernatants were diluted with HNO_3_(1%) to 100 ppb. Finally, the filtered solution was used for elemental concentration analysis via ICP‐MS.

### Phylogenetic analysis

Homologous proteins of nbLTP2 were retrieved from the TAIR (https://www.arabidopsis.org/) and Phytozome (https://phytozome‐next.jgi.doe.gov/) databases. The evolutionary relationships were analysed using MEGA X. The phylogenetic tree was constructed with the following parameters: Jones‐Taylor‐Thornton (JTT) substitution model, Gamma Distributed (G) with four discrete Gamma categories, partial deletion with a 70% site coverage cutoff and Subtree‐Pruning‐Regrafting (SPR) method using a BioNJ initial tree. A strong branch swap filter was applied to enhance the tree's accuracy. Based on the phylogenetic analysis, proteins that fell outside the core tree were excluded. Subsequently, an evolutionary tree for the selected species was obtained from the Timetree database and visualized.

### Statistical analysis

All experiments and data presented here involved three repeats. The data were presented as means and standard deviations. The statistical analysis was performed with SPSS software (version 22.0) using Student's *t*‐test (*0.01 < *p* < 0.05, **0.001 < *p* < 0.01, ***p < 0.001) and one‐way ANOVA test (LSD's test, *p* < 005).

## Author contributions

This study represents an interdisciplinary collaboration. Specifically, J. Wang, X. Wang, C. Liu, X. Zhu and W. Liu conducted biological experiments under the guidance of S. Wang and X. Sun. The materials synthesis and characterization were performed by S. Xiang and Y. Shen under the supervision of X. Ma and J. Huang. All authors contributed to the writing and revision of the manuscript.

## Competing interests

The authors declare no competing interests.

## Supporting information


**Figure S1** EDS of zinc in leaves and stem.
**Figure S2** Response of *N. benthamiana* to exposure to CNC@PDA@Zn^2+^ and (CH_3_COO)_2_Zn for 7d.
**Figure S3** Different expression genes of transcriptome data.
**Figure S4** Effect of CNC@PDA@Zn^2+^ to YoMV infection.
**Figure S5** Heatmap representation of the 100 DEGs between control and CNC@PDA @Zn^2+^.
**Figure S6** Western blot analysis of nsLTP2 protein content.
**Figure S7** Expression of *nsLTP2* after TMV‐GFP infection.
**Figure S8** Expression heat map of chloroplast‐related genes after CNC@PDA@Zn^2+^ treatment.
**Figure S9** Expression of *nsLTP2* in two independent overexpression lines.
**Table S1** The mass fraction of Zn^2+^ on CNC@PDA@Zn^2+^.
**Table S2** Summary of RNA‐seq data and quality control.
**Table S3** Primers used in this study.

## Data Availability

The data that supports the findings of this study are available in the supplementary material of this article.
