# Supplementary material for: High‐permeability cellulose nanocrystals mediate systemic zinc redistribution through nsLTP2‐dependent immune potentiation in plants
Source: Plant Biotechnol J. 2025 Jun 26;23(9):4175–90. doi: 10.1111/pbi.70230 (PMC12392954; doi:10.1111/pbi.70230)
Supplement: Supplementary file 1 — Figure S1 EDS of zinc in leaves and stem. Figure S2 Response of N. benthamiana to exposure to CNC@PDA@Zn2+ and (CH3COO)2Zn for 7d. Figure S3 Different expression genes of transcriptome data. Figure S4 Effect of CNC@PDA@Zn2+ to YoMV infection. Figure S5 Heatmap representation of the 100 DEGs between control and CNC@PDA @Zn2+. Figure S6 Western blot analysis of nsLTP2 protein content. Figure S7 Expression of nsLTP2 after TMV‐GFP infection. Figure S8 Expression heat map of chloroplast‐related genes after CNC@PDA@Zn2+ treatment. Figure S9 Expression of nsLTP2 in two independent overexpression lines. Table S1 The mass fraction of Zn2+ on CNC@PDA@Zn2+. Table S2 Summary of RNA‐seq data and quality control. Table S3 Primers used in this study. [file PBI-23-4175-s001.docx]

**
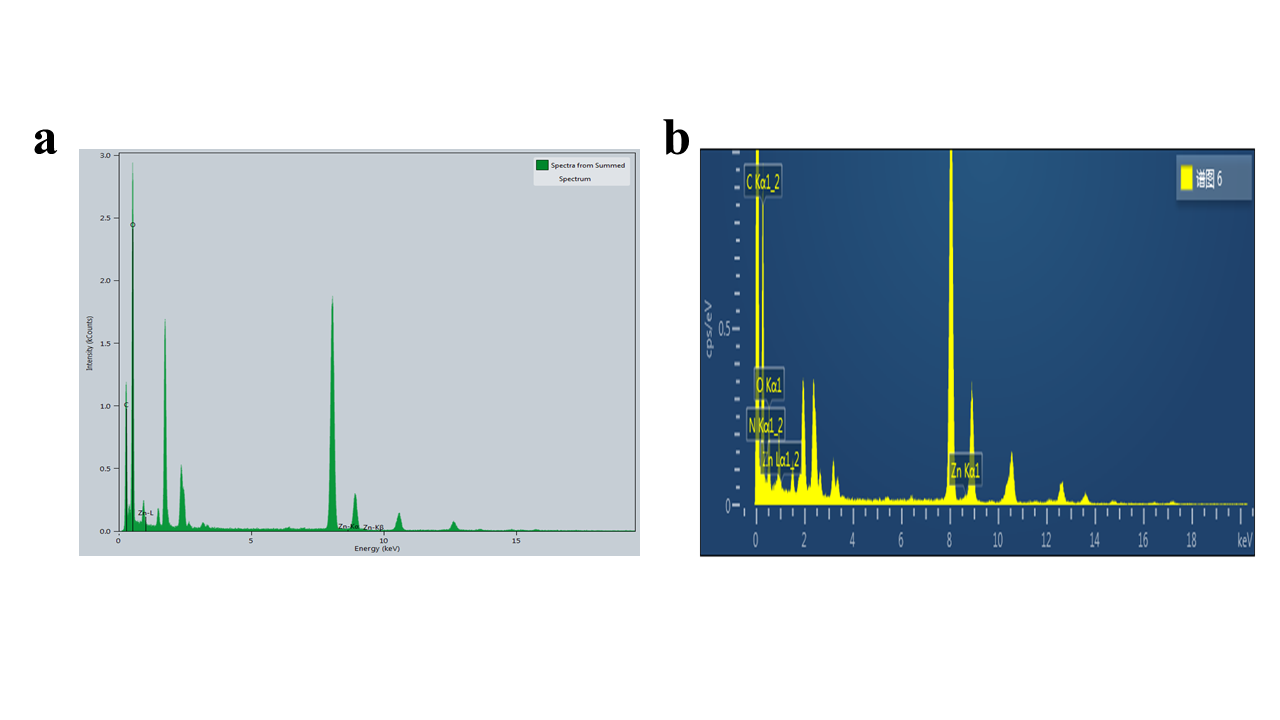
**

**Fig S1** **EDS of Zinc in leaves and stem.** (a) EDS results show the CNC@PDA@Zn^2+^ in leaves. (b) EDS results show the CNC@PDA@Zn^2+^ in stem.


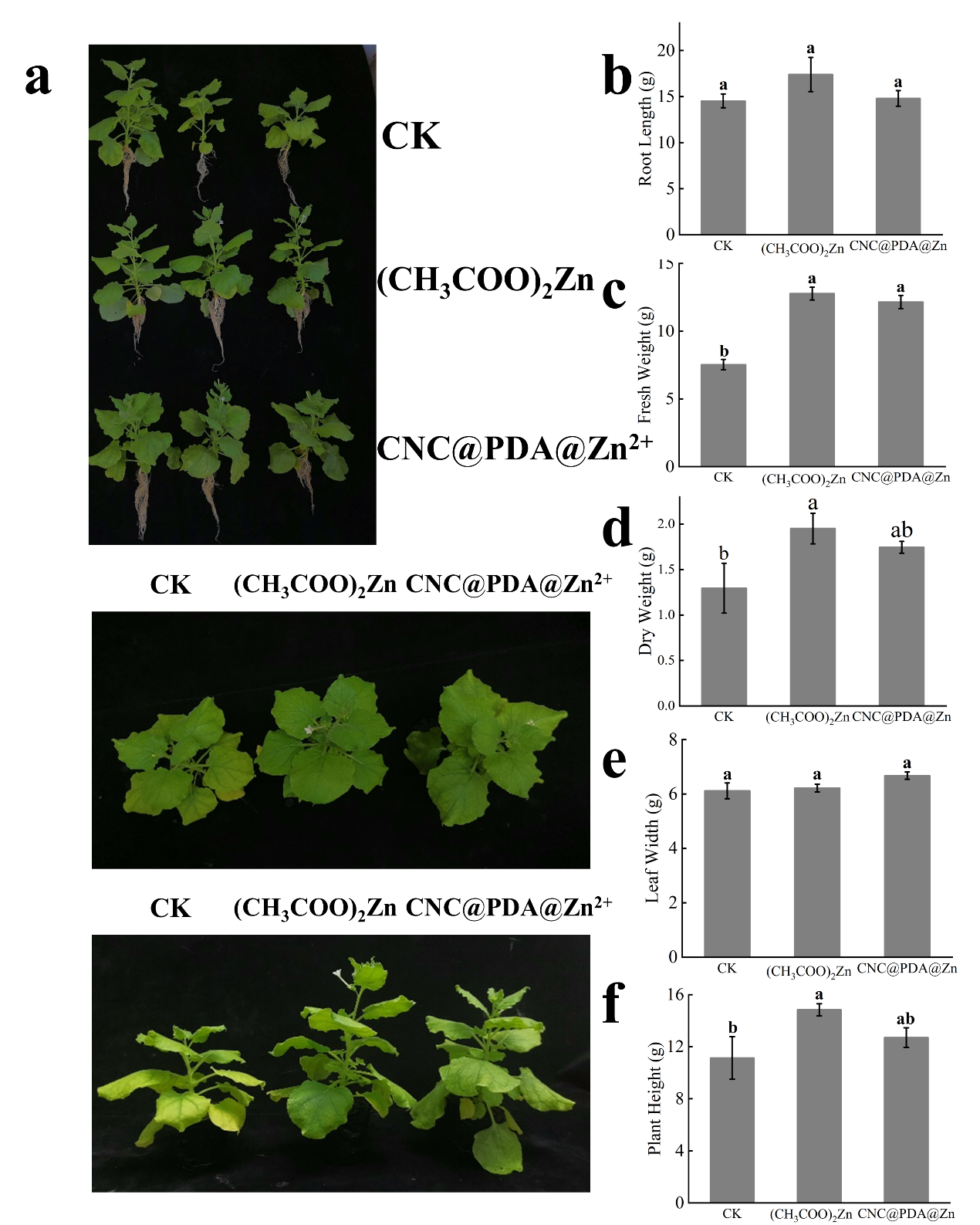


**Fig S2 Response of tobacco plants to exposure to CNC@PDA@Zn^2+^ and (CH_3_COO)_2_Zn for 7d.** (a) Phenotypic images of tobacco upon exposure to CNC@PDA@Zn^2+^ and (CH_3_COO)_2_Zn. (b)-(f) Root length, fresh weight, dry weight, leaves width and plant height after foliar treatment with CNC@PDA@Zn^2+^ and (CH_3_COO)_2_Zn. Mean values displayed in each bar followed by different letters are significantly different according to LSD’s multiple range test (*p* < 0.05).

**
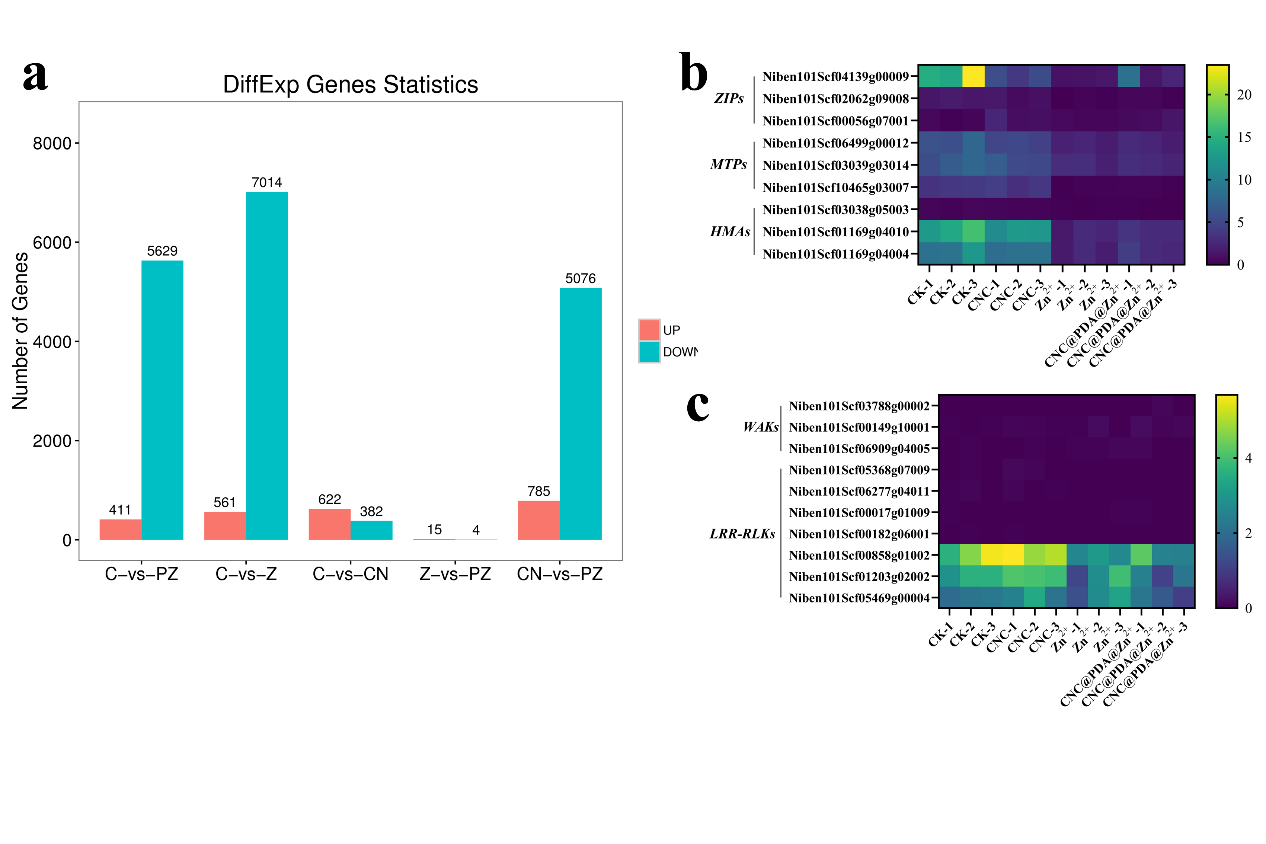
 Fig S3 Different expression genes.** (a) Different expression genes in C-Z, C-PZ, C-CN, CN-PZ, Z-PZ different group. (b) DEGs related to Zn^2+^ transport protein family. (c) DEGs related to cellulose-recognizing receptors. C: CK, PZ: CNC@PDA@Zn^2+^, Z: (CH_3_COO)_2_Zn, CN: CNC.

**
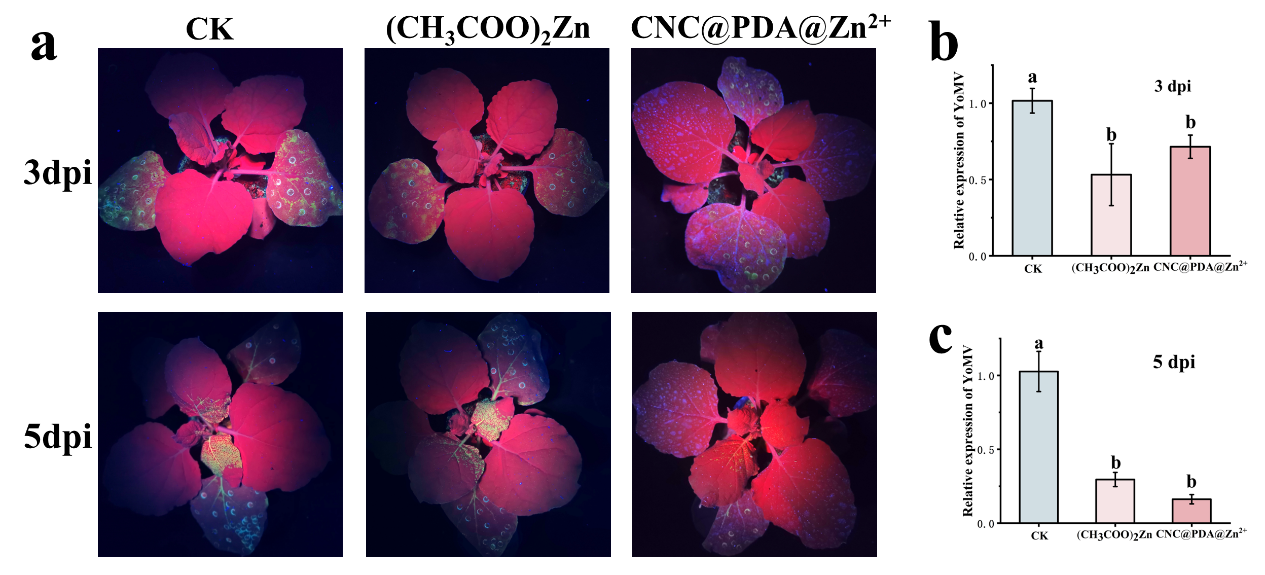
Fig S4** **Effect of CNC@PDA@Zn^2+^ to YoMV infection.** (a) antivirus activity of (CH_3_COO)_2_Zn (44μg/mL) and CNC@PDA@Zn^2+^(1600μg/mL). CK as the control group, the concentration of Zn^2+^ in 44 μg/mL (CH_3_COO)_2_Zn was the same as the concentration of Zn^2+^ loaded in 1600 μg/mL CNC@PDA@Zn^2+^. (b-C) YoMV expression at 3 dpi and 5 dpi. Mean values displayed in each bar followed by different letters are significantly different according to LSD’s multiple range test (p < 0.05). Vertical bars indicate standard errors (n = 5).

**
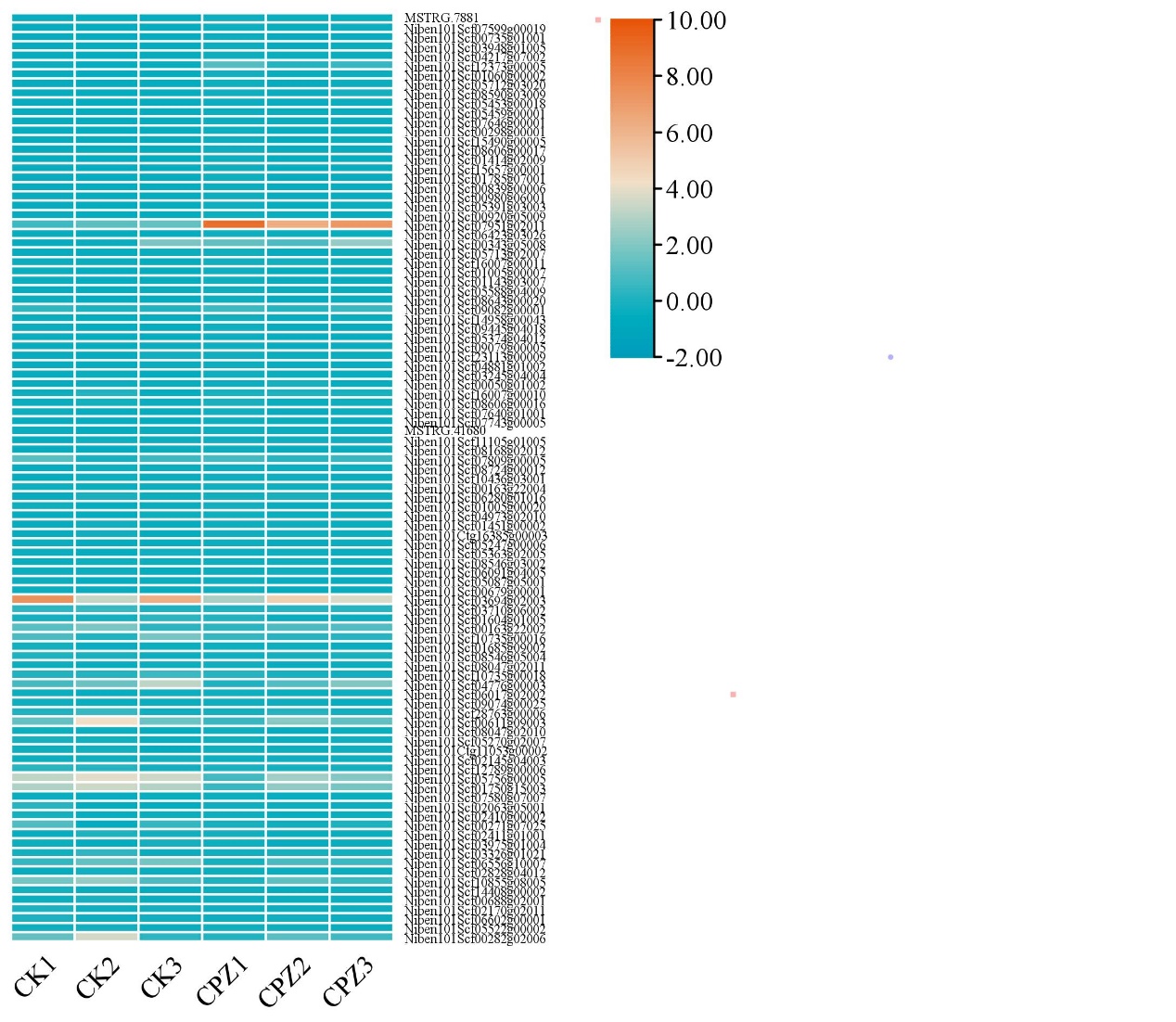
Fig S5** **Heatmap representation of the expression of 100 DEGs between control and CNC@PDA @Zn^2+^.** C: CK, CPZ: CNC@PDA@Zn^2+^.

**
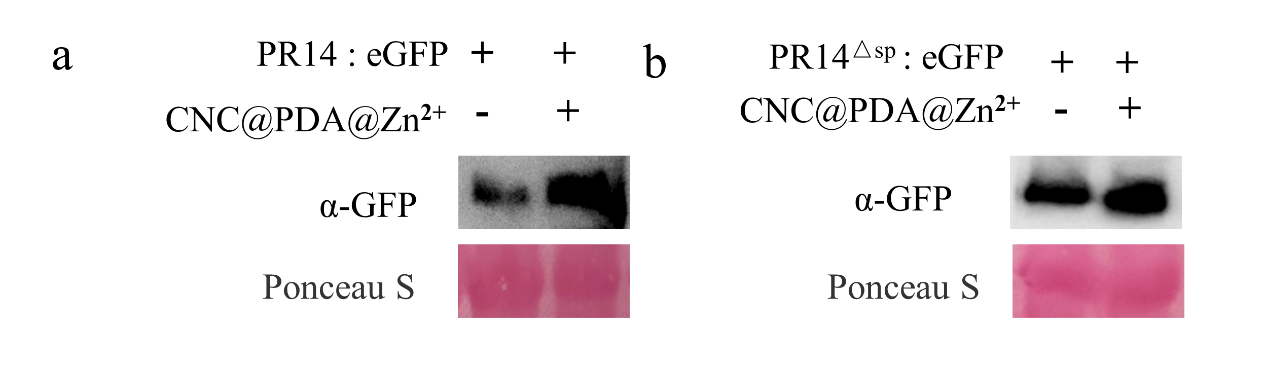
Fig S6** **Western blot analysis of nsLTP2 protein content**. (a) Western blot analysis of nsLTP protein content. (b) Western blot analysis of nsLTP ^△sp^ protein content.


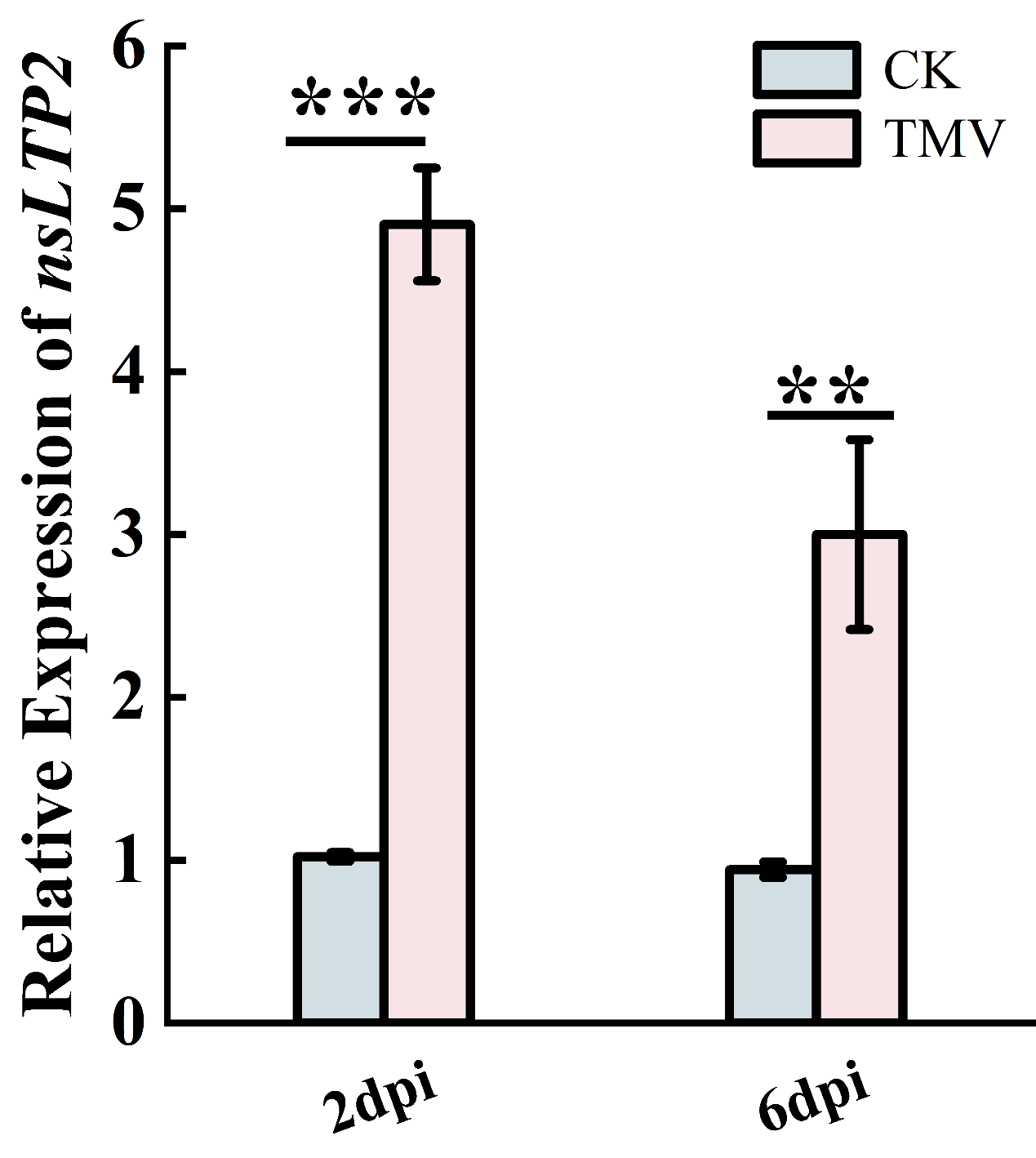
**Fig S7** **Expression of *nsLTP2* after inoculate TMV-GFP at 2 dpi and 6 dpi.** The samples of 2 dpi were the inoculated leaves and the samples of 6 dpi were the systemic leaves.


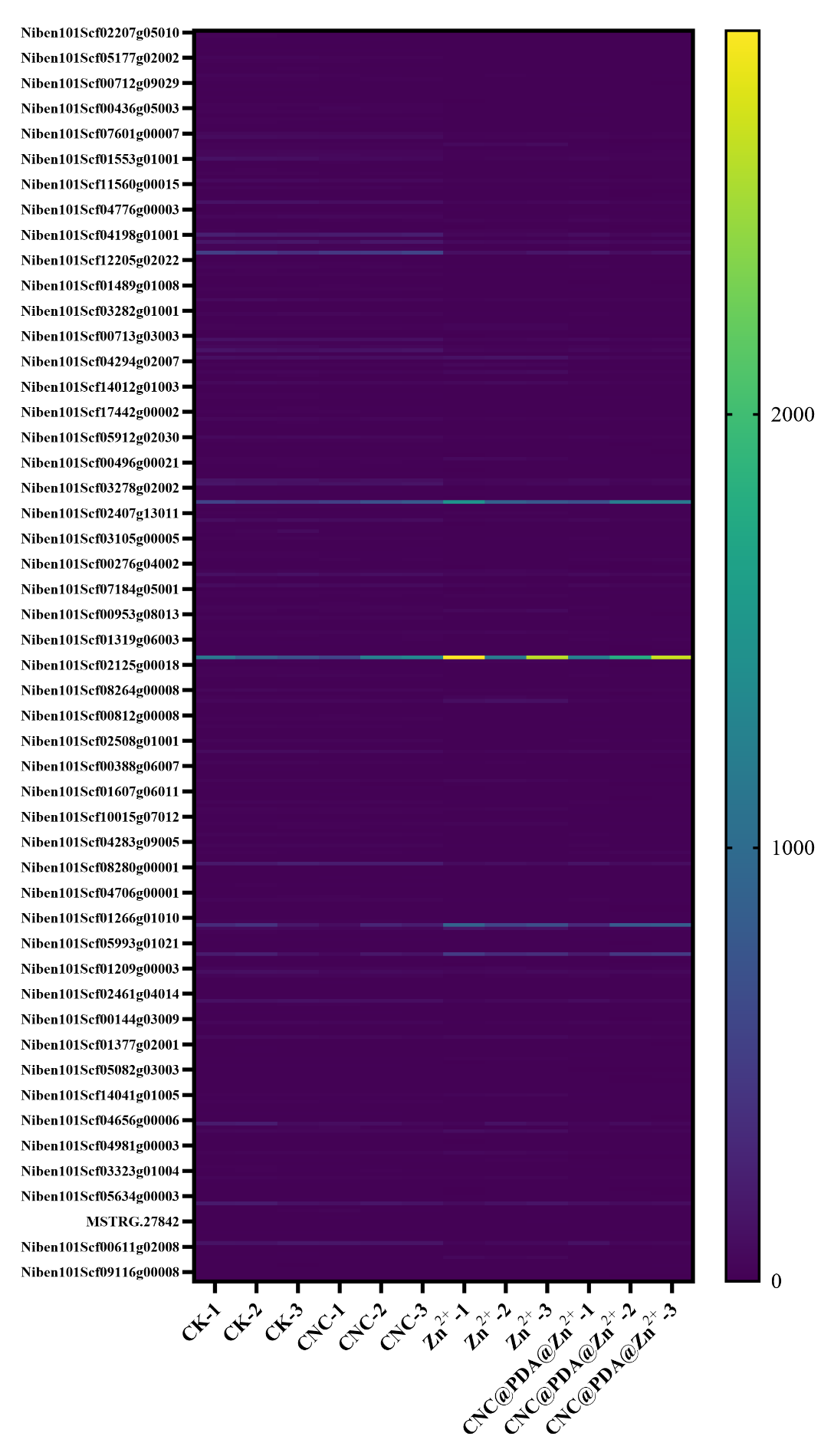


**Fig S8** Expression heat map of chloroplast-related genes after CNC@PDA@Zn^2+^ treatment.


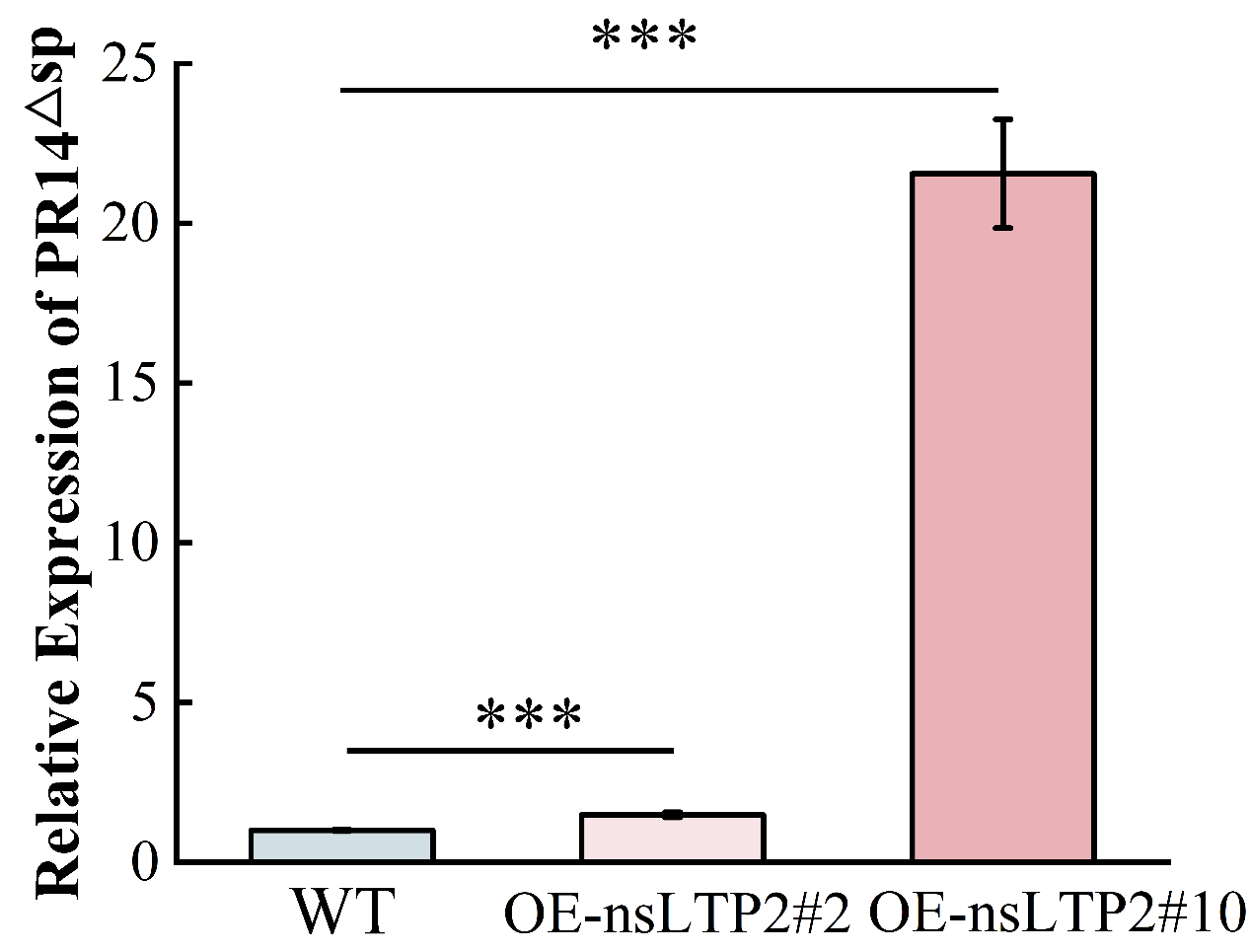


**Fig S9** Expression of *nsLTP2* in two independent overexpression lines.

**Table S1** The mass fraction of Zn^2+^ on CNC@PDA@Zn^2+^

| sample |  | Zn  (213.857 nm)/ppm | Weight of Zn^2+^ in 10 mL solution/mg | Weight of CNC in 10 mL solution/mg | Concentration of Zn^2+^ | Weight percent of zinc (wt%)/% |
| --- | --- | --- | --- | --- | --- | --- |
| 1 |  | 23.36 | 0.2336 | 21.0455 | 0.0111 | 1.109976 |
| 2 |  | 23.58 | 0.2358 | 21.6241 | 0.010905 | 1.09045 |
| 3 |  | 24.04 | 0.2404 | 22.1421 | 0.010857 | 1.085715 |
| 4 |  | 23.88 | 0.2388 | 21.3241 | 0.011199 | 1.11986 |
| 5 |  | 24.25 | 0.2425 | 21.3059 | 0.011382 | 1.138182 |
| 6 |  | 23.36 | 0.2336 | 21.0455 | 0.0111 | 1.109976 |

**Table S2** Summary of Sequencing Data Statistics and Quality Control

| Sample | Raw reads | Clean reads | GC (%) | Q30 (%) | Total mapped reads (%) | Unique Mapped reads (%) |
| --- | --- | --- | --- | --- | --- | --- |
| C-1 | 51315686 | 51248582 | 45.33 | 94.52 | 97.98 | 87.85 |
| C-2 | 52753948 | 52691178 | 45.54 | 94.41 | 97.96 | 87.93 |
| C-3 | 47431746 | 47361844 | 45.10 | 94.29 | 97.91 | 88.51 |
| PZ-1 | 45231642 | 45163754 | 45.79 | 94.29 | 97.90 | 87.65 |
| PZ-2 | 55532484 | 55468572 | 46.34 | 94.46 | 97.91 | 87.00 |
| PZ-3 | 47765010 | 47710136 | 46.60 | 94.48 | 98.09 | 86.05 |
| Z-1 | 49842008 | 49789618 | 46.92 | 94.28 | 97.93 | 85.62 |
| Z-2 | 56271726 | 56203736 | 46.29 | 94.16 | 98.08 | 87.40 |
| Z-3 | 42497600 | 42460498 | 47.26 | 94.94 | 98.07 | 85.83 |
| CN-1 | 45519812 | 45460260 | 44.86 | 94.08 | 97.84 | 89.43 |
| CN-2 | 46635806 | 46581580 | 45.67 | 94.44 | 98.08 | 87.50 |
| CN-3 | 49416894 | 49366580 | 45.75 | 94.45 | 97.97 | 87.07 |

Note: C means CK control, Z means Zn^2+^ control, CNC means CNC-treated plants; PZ means CNC@PDA@Zn^2+^ treated plants

**Table S3** Primer sequence used in this work

| Primers names | Primer sequence | |
| --- | --- | --- |
| q*NbActin*-F | | 5'-CTTGAAACAGCAAAGACCAGC-3' |
| q*NbActin*-R | | 5'-CATCCTATCAGCAATGCCCG-3' |
| q*NbTMV-MP*-F | | 5'-GACCTGACAAAAATGGAGAAGATCT-3' |
| q*NbTMV-MP*-R | | 5'-GAAAGCGGACAGAAACCCGCTG-3' |
| q*nsLTP2*-F | | 5'- GGTGTTAAGCGTCTGTTGGG -3' |
| q*nsLTP2*-R | | 5'- AGTGGTGGGACTGATCTTGT -3' |
| q*NPR1*-F | | 5'-TAGCGTATTGCGATGCAAAG-3' |
| q*NPR1*-R | | 5'-TAGTGAGCCTCTTGGCGATT-3' |
| q*PR1*-F | | 5'- ATGGTCAATACGGCGAAAAC -3' |
| q*PR1*-R | | 5'- CCTAGCACATCCAACACGAA -3' |
| q*PR2*-F | | 5'- CAACCCGCCCAAAGATAGTA-3' |
| q*PR2*-R | | 5'- TCCAAAAGGGCATCAAAAAG-3' |
| TRV:PR14-F | | 5'- GCTCTAGAAGATTGCTTG -3' |
| TRV:PR14-R | | 5'- CCGCTCGAGGCATGCAGTTTTGC -3' |
| pArt27-PR14: eGFP-F | | 5'-TTGGAGAGGACACGCTCGAGATGGAAATAGCTGGCAAGAT -3' |
| pArt27-PR14: eGFP -R | | 5'- CCTCGCCCTTGCTCACCATCTCGAGCTGGACCCTGGAGCAGTCAG -3' |
| pArt27-PR14: eGFP^Δsp^-F | | 5'- TTGGAGAGGACACGCTCGAGATGCTGATGAATTGCGGCCAGG -3' |
| pArt27-PR14: eGFP^Δsp^-R | | 5'- CCTCGCCCTTGCTCACCATCTCGAGCTGGACCCTGGAGCAGTCAG -3' |
